# Quantitative RT-PCR based platform for rapid quantification of the transcripts of highly homologous multigene families and their members during grain development

**DOI:** 10.1186/1471-2229-12-184

**Published:** 2012-10-09

**Authors:** Agnieszka Kaczmarczyk, Steve Bowra, Zoltan Elek, Eva Vincze

**Affiliations:** 1 Department of Genetics and Biotechnology, Aarhus University, Research Centre Flakkebjerg, Forsøgsvej 1, Slagelse, DK-4200, Denmark; 2 Verzyme (UK) Ltd., Plas Gogerddan, Aberystwyth, Wales, SY23 3EB, United Kingdom; 3 MTA-ELTE-MTM Ecology Research Group, Biological Institute, Eötvös Loránd University, Pázmány Péter sétány 1C, Budapest, H-1117, Hungary

**Keywords:** SYBR Green, High homology multigene families, Transcript abundance, Hordeins

## Abstract

**Background:**

Cereal storage proteins represent one of the most important sources of protein for food and feed and they are coded by multigene families. The expression of the storage protein genes exhibits a temporal fluctuation but also a response to environmental stimuli. Analysis of temporal gene expression combined with genetic variation in large multigene families with high homology among the alleles is very challenging.

**Results:**

We designed a rapid qRT-PCR system with the aim of characterising the variation in the expression of hordein genes families. All the known D-, C-, B-, and γ-hordein sequences coding full length open reading frames were collected from commonly available databases. Phylogenetic analysis was performed and the members of the different hordein families were classified into subfamilies. Primer sets were designed to discriminate the gene expression level of whole families, subfamilies or individual members. The specificity of the primer sets was validated before successfully applying them to a cDNA population derived from developing grains of field grown *Hordeum vulgare* cv. Barke. The results quantify the number of moles of transcript contributed to a particular gene family and its subgroups. More over the results indicate the genotypic specific gene expression.

**Conclusions:**

Quantitative RT-PCR with SYBR Green labelling can be a useful technique to follow gene expression levels of large gene families with highly homologues members. We showed variation in the temporal expression of genes coding for barley storage proteins. The results imply that our rapid qRT-PCR system was sensitive enough to identify the presence of alleles and their expression profiles. It can be used to check the temporal fluctuations in hordein expressions or to find differences in their response to environmental stimuli. The method could be extended for cultivar recognition as some of the sequences from the database originated from cv. Golden Promise were not expressed in the studied barley cultivar Barke although showed primer specificity with their cloned DNA sequences.

## Background

Cereal-derived products constitute a major part of human and livestock diets. In 2009 the annual production of all cereals exceeded 2,400 million tonnes [1]. Assuming an average protein content of 10%, over 240 million tonnes of grain protein was harvested in 2009. The average protein content of cereals indicates that relatively small differences exist within and between species and that these can be amplified by environmental factors. For example the protein content on a dry weight basis is in the range of 10–15% in wheat [2]; 8–15% for barley [3] or 9.1-13.3% for rye [4].

Improvement in complex traits such as cereal yield and quality through plant breeding and the associated molecular and biochemical tools is vital to keep pace with population growth and nutritional requirements. Considerable effort has been directed at developing a range of genomic DNA markers with the goal of supporting marker assisted plant breeding. The value of anonymous genetic markers such as random DNA markers (SSRs, AFLPs, RFLPs etc.) depends on the known linkage between marker and target locus alleles [5]. In contrast, “functional markers” (FMs) are derived from polymorphic sites within genes, quantitative trait nucleotides (QTN) or quantitative trait insertion – deletion mutations (QTINDEL) and have significant potential to translate genomic technologies into improved crop varieties. Once genetic effects have been assigned to functional sequence motifs, FMs can be used for fixation of beneficial alleles [6,7]. Syvänen [8] has described five phases of functional marker development (1) functionally characterised genes, (2) allele sequences from such genes, (3) identification of polymorphic, functional motifs affecting plant phenotype within these genes, (4) validation of associations between DNA polymorphisms and trait variation, and (5) conversion into technical assays using, e.g., any of the single nucleotide polymorphism (SNP) or INDEL detection technologies. To date, only a limited number of genes have been isolated for functional markers development in wheat [9] and to the authors knowledge there are no reports of functional marker development with respect to storage proteins in barley, the focus of our research.

The genetic control of grain protein content in barley has been reviewed by Ullrich [10] and the trait is clearly polygenic, with quantitative trait loci (QTLs) mapping onto all seven chromosomes. Hordeins, the main storage proteins of the barley endosperm are encoded by a single multigenic locus located on chromosome 5 and are divided into four groups: B-hordein (sulphur-rich), C-hordein (sulphur-poor), γ-hordein (sulphur-rich) and D-hordein (high molecular weight), distinguishable by their electrophoretic mobility and amino acid composition. The four groups are encoded by the genes: *Hor2* (B-fraction), *Hor1* (C-fraction), *Hor3* (D-fraction) and *Hor5* (γ-fraction), located on barley chromosome 5 (1 H). The B-hordeins account for 70-80% (mol wt 35–46 kDa); the C-hordeins 10–20% (mol wt 55–75 kDa); the D hordeins 2–4% (mol wt 100 kDa) and the γ-hordeins amount <5% (35–46 kDa) of the total hordein fraction [3]. The *Hor2* gene family encoding the B-hordeins and *Hor1* loci encoding C-hordeins are thought contain between 20–30 genes per haploid genome [11,12]. The D-and γ-hordein groups, encoded by the *Hor3* and *Hor5* loci respectively [3,13], are minor components and little is known of the extent of polymorphism of the genes although their products extensively studied [14,15].

The variation in barley storage protein allelic complement and the associated contribution to the storage protein profiles both in terms of amino acid and polypeptide composition provides the bases for the observed significant variation within and between that barley cultivars and wild relatives with respect to the number and type of storage proteins/polypeptides [16,17].

To address the need for studying the expression patterns of hordein genes, a rapid qRT-PCR screening method was developed and verified. Our work provides a tool for identifying presence of the storage protein alleles and their expression in developing barley grain. We assume that it can be also used to study changes in a response to different environmental conditions or be assistance in researching genetic markers and more specifically, functional markers, in barley. Although the impact of cultivar-specific polymorphism could be considered as a limitation of the method, at the same time it could be used to highlight the inherited problems of the primer design when database sequences that originate from many different cultivars are used.

## Results

### Hordein sequence analysis and primer design

The first step toward developing the high throughput platform was to curate all known hordeins sequences from EMBL Nucleotide Sequence Database (EMBL), DNA Data Bank of Japan (DDBJ), GenBank at the NCBI (GenBank) and HarvEST databases. Incomplete, partial and EST sequences were filtered out of the collection to ensure that only accessions that coded for the full proteins were used. All the accession numbers of the genes used in this study can be found in Table 1.

**Table 1:** Hordein and selected housekeeping genes

**Gene**	**Gen Bank**	**Barley cultivar**	**Size of the coding region (bp)**	**Amino acid residues**
	**Accession number**			
B-hordein	JQ867081	Golden Promise	804	267
B-hordein	JQ867082	Golden Promise	837	278
B-hordein	JQ867083	Golden Promise	861	286
B-hordein	JQ867084	Golden Promise	762	253
B-hordein	JQ867085	Golden Promise	933	310
B-hordein	JQ867086	Golden Promise	855	284
B-hordein	JQ867087	Golden Promise	840	279
B-hordein	JQ867088	Golden Promise	933	310
B-hordein	JQ867089	Golden Promise	834	277
B-hordein	GQ342970	Z26	798	265
B-hordein	GQ342971	Z26	798	265
B-hordein	GQ342972	Z26	798	265
B-hordein	GQ342973	Z26	798	265
B-hordein	GQ342975	Z26	798	265
B-hordein	GQ342976	Z26	798	265
B-hordein	DQ267478	ZQ7239	798	265
B-hordein	DQ826387	ZQ148	873	265
B-hordein	X03103	Sundance	882	293
B-hordein	DQ148297	XQ053	903	300
B-hordein	X87232	Carlsberg II	816	271
B-hordein	JQ859915	Barke	873	290
B-hordein	JQ859916	Barke	816	271
B-hordein	JQ859917	Barke	783	260
B-hordein	JQ867073	Barke	885	294
B-hordein	JQ867074	Barke	873	290
B-hordein	JQ867075	Barke	873	290
B-hordein	DQ267479	Aba-zhangla	894	297
B-hordein	DQ178602	Aba-siqing	873	290
B-hordein	X53690	Moskovsky 3	873	290
C-hordein	S66938	Odessky 46	1017	338
C-hordein	X60037	Bomi	867	288
C-hordein	JQ867090	Barke	909	302
D-hordein	AY268139	Morex	2274	757
D-hordein	D82941	Haruna Nijo	2124	707
D-hordein	JQ867076	Golden Promise	2244	747
D-hordein	JQ867077	Golden Promise	2184	727
D-hordein	JQ867091	Barke	2244	747
γ1-hordein	X13508	Carina	918	305
γ1-hordein	AJ580585	Riso 56	768	255
γ1-hordein	JQ867078	Barke	918	305
γ1-hordein	JQ867079	Golden Promise	888	295
γ3-hordein	X72628	Carlsberg II	855	284
γ3-hordein	JQ867080	Golden Promise	918	305
Actin	AY145451	Himalaya	1133	377
Ubiquitin family	AK249354	Haruna Nijo	471*	156*
Protein translation factor (GOS2)	AK252057	Haruna Nijo	348**	115**

* The length of *Arabidopsis thaliana* protein (gb|AAF24594.1).

** The length of *Oryza sativa* protein (gb| EF575854.1).

**Figure 1:**
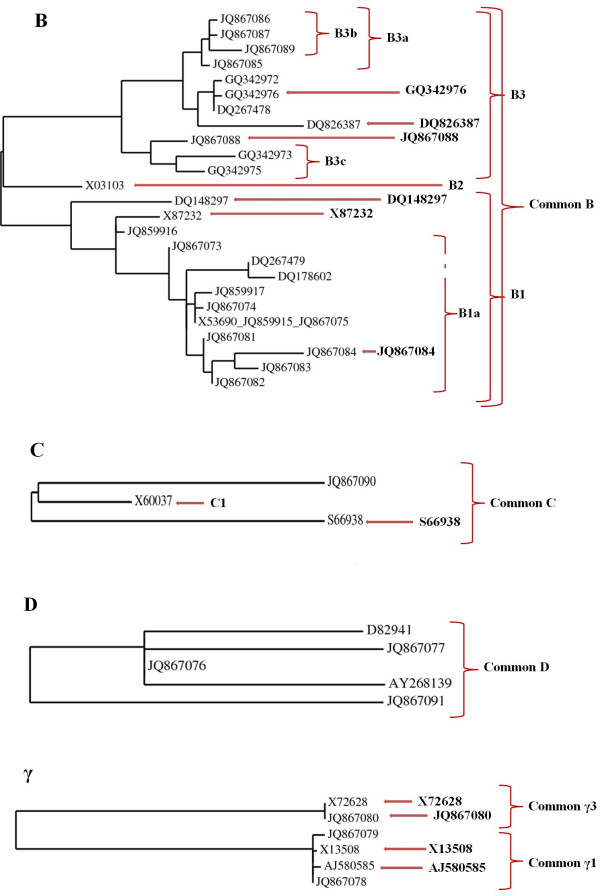
**Phylogenetic trees of B-, C-, D- and γ- hordeins with designed primers.** Brackets and arrows indicate primers recognizing families, subfamilies, subgroups and individual members of the multigene families.

Hordein genes are characterised by repeated sequence motifs and the impact and evolution of this feature has been extensively reviewed [3,18,19]. A full discussion of the impact of repeats on evolutionary analysis is beyond the scope of this work. However a typical phylogenetic analysis of sequence data involves five distinct steps: (a) data collection, (b) inference of homology, (c) sequence alignment, (d) alignment trimming, and (e) phylogenetic analysis [20]. As part of the ‘alignment trimming’ steps a core sequence was chosen to improve the quality of the multiple sequence alignment but also assist the primer design. The core sequences were selected by cutting off most of the repetitive regions. The 29 different B-hordein alleles (full lengths are between 762–933 bp) were trimmed to 596 to 609 bp, while the chosen “core” sequences of the three different C-hordein alleles were 640 bp (full lengths are between 867–1017 bp), 1220 bp for the five D-hordein alleles (full length sequences are between 2124–2274 bp) and 649 bp for the different γ-hordein alleles (full lengths are between 768–918 bp) (Additional file 1). The ‘core’ sequences were used to create multiple sequence alignment and design the primers. The phylogenetic analysis was performed on the full length clones, core sequences and proteins, all producing the same arrangements within the families. The results of using the protein sequences were visualised in unrooted phylogenetic tree (Figure 1). 

The sequence identity of hordein alleles is very high: 84 to 99% for B-, 80 to 92% for C-, 99% for D-, from 98 to 99% for γ1 and 94% for γ3-hordeins, therefore designing specific primers for groups, subfamilies and individual members are challenging. Primers were designed to recognise whole groups and where it was appropriate subfamilies or individual members of the gene families (Figure 1; Table 2).

**Table 2:** Primer sets for B-, C-, D- and γ-hordein transcripts and reference genes

**Name**	**Primer sequence forward**	**Primer sequence reverse**
**Common B**	TTTCCAACAACCTCAACCACA	GTAGGGTACGCAGCGCAAT
B1	GCAAGAACAACCCCAACAGTC	GTAGGGTACGCAGCGCAAT
B1a	CCAGCAACTGCCGCAAATCT	CAACTGTTGTTGGGTTTGGGAT
DQ148297	CCACTCCAGCTAGCTCAC	TGCCGAATGGAAGTATGCG
X87232	GGGCTACAACAACCAATTCTG	CCTTGTGGGAGTGGTGTTG
JQ867084	CCAACAGTCGGTCCAAGGA	CATTGTCCAACTTTCTCCTGC
B2	CCGCAGCAAGTCGGACAA	CCTGTTGTTGTTGACCAAC
B3	GTGCAATCGTCTACTCTATCG	GGGAGACACCTTGGACCAAT
B3a	CTATCGTCCTGCGAGAACAAT	CCGACTTGTTGCTGTTGTG
B3b	ACGTATTGCAAGGTCGCAG	CGGCAGTTGTTGGCAACAC
B3c	AAACAACAGCAAGTGCCACAT	TCGCCTCAAGCTGAGCTAG
DQ826387	CCTCAACCACAACAAGTTGGC	CTGTACGACGGCACATTAACAC
GQ342976	CTCCTACAAGAACAACAAGAC	CCTTGCATGGGTTTAGCTGC
JQ867088	AACAACAGTCGCAGCTACAT	TTCAAGCTGAGCTAGCTGGA
**Common C**	TAATTCCCCAGCAACCTCAA	CCATACTCCAGATGGTTTGTTG
C1	TCAACCAGTCCCCCAGCA	CTTGTTGGGGTTGCGGTT
S66938	CCTCAACAACCATTTCCCCT	AAATGGTTGTTGTGGTTGCCA
**Common D**	CACCGTGTCTCTGCACCATG	TGCCGTAGTACAACTCGTTGG
**Common γ1**	CAACCGCAACAACTAGCTCA	CACCAACAAATGGTGCTTTG
AJ580585	CCAACAACAACTGAATCCGTA	TTGCAGGCAACATTGTTGCA
X13508	CCTGTGTCATTGTTATCGTACA	CGACAACTGCTCTGTTGCAC
**Common γ3**	GGTTGGGTCATTGGTGATTC	AGCAATAAGGTGGGACATGC
X72628	AGCAAATATCAATGAGCAG	GAGATTGGACAAAACCATGAC
JQ867080	AGCAAATATCAATGAGCAA	GAGATTGGACAAAACCATGAT
**Actin**	CCTCAGTTGAGAAGAGCTACG	TCTGCGCCAATCGTGATC
**Ubiquitin**	TCAAGGTGAAGACACTTACTGG	CATAGATGAGCCTCTGTTGAAC
**PTF**	CTATGTGCATGTGCGTGTC	CTTGAGAATCTTGTTGTAGCTG

B-hordein represents 70-80% of the hordein storage protein, as such is the most significant class of hordeins in terms of amount protein found in the mature grain [3]. The phylogenetic analysis of the B-hordein sequences divided the population into two major subfamilies: B3 and B1, and a minor subfamily B2, these subfamilies could be further subdivided in to groups according to their sequence differences (Figure 1). Primer sets were designed to recognise the whole gene family (Common B primer set); the subfamilies (B1-, B2- and B3-), subgroups of subfamilies (B1a; B3a; B3b; B3c) and individual members (GQ342976, DQ826387, JQ867088, DQ148297, X87232, JQ867084) (Figure 1; Table 2).

In mature barley grains C-hordein accounts for approximately 10-20% of the total hordein protein [3]. However, to date, only three full length sequences have been deposited on the databases from three different cultivars (Table 1). Phylogeny analysis of the C-hordein sequences identified two subfamilies and the two groups showed MW differences as well (Figure 1; Table 1). A common C-hordein primer set was designed to study the expression of the whole family (Common C primer set) and in addition primers specific for the subfamilies were also created (C1, S66938) (Figure 1; Table 2).

D-hordein accounts for less than 5% of total grain protein in the mature grain [3]. Trawling the databases revealed five highly similar coding sequences corresponding to D-hordein derived from four different cultivars. Given the sequence architecture of D-hordein we designed a common primer set for all the genes. The phylogenetic analysis generated two subfamilies, which we annotated D1 and D2 but were unable to design primers to distinguish the specific alleles as the differences among the sequences were in the number of repeats (Figure 1; Table 1; Table 2). It is suggested that an alternative method such as standard RT-PCR could be used to differentiate the expression of the D-hordein alleles [21].

Gamma-hordeins are represented by a small group of protein, their contribution to the total grain protein has not been precisely determined [3]. We obtained six sequences from five cultivars and these were apportioned into two subfamilies, γ1 and γ3 by phylogenetic analysis (Figure 1; Table 1). As the coding sequences of two subfamilies are very different we were not able to design a common primer sets for the family. Common primers were designed for γ1- and γ3-subfamilies and four specific primers were designed for individual subgroups (Figure 1; Table 2).

### Quantitation of the DNA concentration by standard curve using qRT-PCR reaction

**Figure 2:**
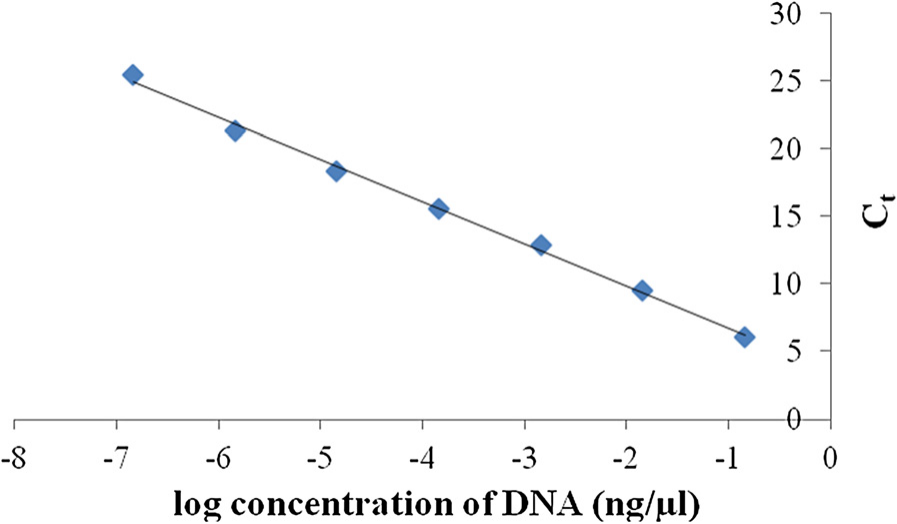
**Standard curve for the actin DNA.** The data presented as C_t_ values versus DNA concentration (y = −3.1136x + 3.6102; R^2^ = 0.9968). The calculation based on three replicates and presented as means ± SD (error bars are not visible due to their small size).

In order to construct standard-curves, serial dilutions of known amounts of cloned actin DNA was-amplified in the qRT-PCR reactions. Actin DNA was diluted from 1.44 ng/μl to 1.44 fg/μl (10 times dilution series) (Figure 2). The standard was included in every plate and the PCR efficiencies were between 94.25% and 109.49%. From the dilution curves and calculated PCR efficiencies the range of reliable and acceptable C_t_ values was established between 5 and 28. Based on the slopes for hordein and reference primers we selected the optimal cDNA concentrations to calculate expression level of hordein transcripts.

### Validation of primer specificity and standard curve by qRT-PCR reaction using cloned DNA targets

**Figure 3:**
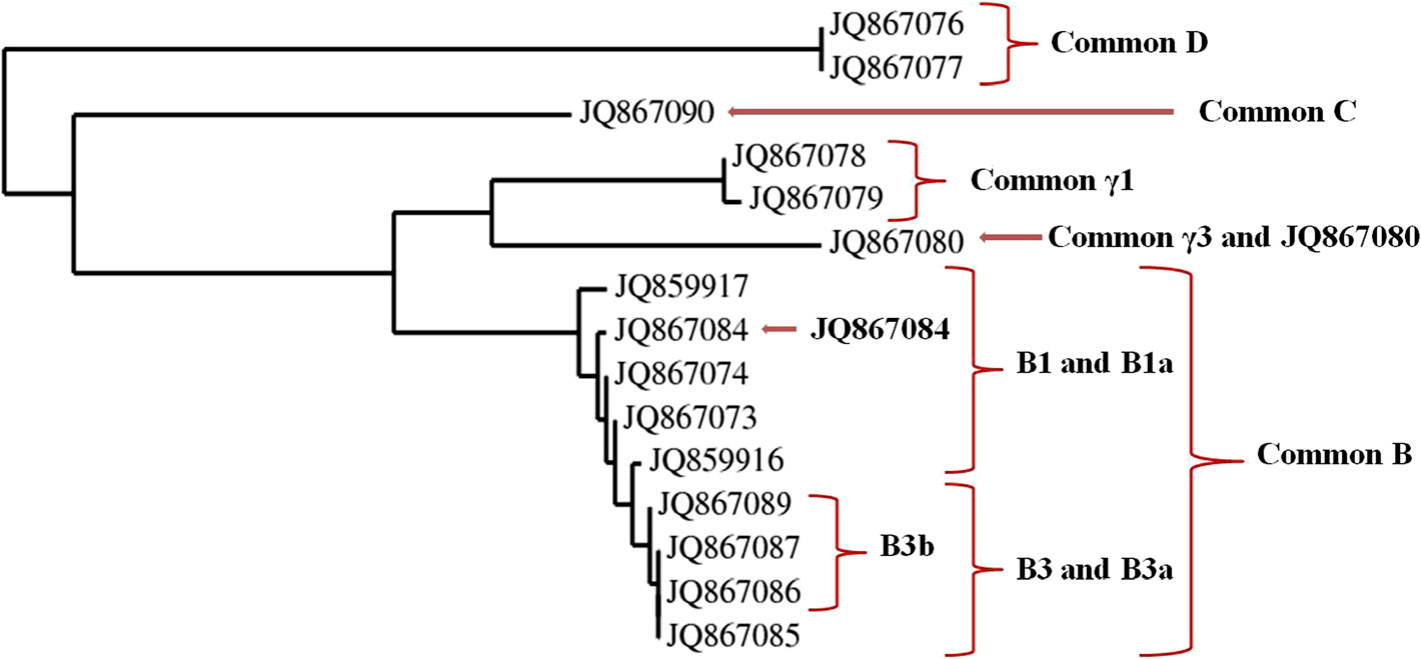
**Phylogenetic tree of hordeins used in the validation of actin standard curve.** Brackets and arrows indicate primers recognizing families and subfamilies, subgroups and individual members of the multigene families.

In order to validate the efficacy of allelic specific primers to accurately discriminate between alleles within a cDNA population it was necessary to demonstrate the primer specificity on the DNA template of the corresponding allele. We have 15 different hordein DNA clones representing 9 B-, 1 C-, 2 D-, and 3 γ-hordein alleles. Each primer set, which had been designed towards a specific allele or alleles was tested both on the DNA of the target allele but also in the presence of non-target allelic DNA. A primer set was designated as specific when only target gene or genes were amplified. Validated primers, specific to the selected hordein groups are presented in Table 2 and in the Additional file 1. The phylogenetic relationship and the primers sets used are illustrated in Figure 3.

We used the above mentioned 15 different hordein DNA clones to validate the qRT-PCR with actin standard curve (Figure 2). The validation experiment was performed by adding same amount of DNA to the DNA pool from the individual clones and their amol/μl concentrations were calculated. For example, common B group had 9 individual members with the total 5.16 amol/μl DNA in the pooled DNA sample and the DNA concentration calculated from the C_t_ value was 5.71 ± 0.18 amol/μl (Table 3). B1 and B3 subgroups showed high similarities between the added and calculated DNA concentrations: 2.94 vs. 2.92 ±0.11 and 2.21 vs. 2.41 ± 0.61 amol/μl, respectively. Furthermore the calculated B1 and B3 values added up to the total calculated common B values 5.33 ±0.36 vs. 5.71 ± 0.18 amol/μl (Table 3). The method was working as well when individual members like the B1- (JQ867084) or γ1- (JQ867080) hordein were evaluated from the pool. Overall, we obtained similar values when the target DNA concentrations were measured versus calculated from the C_t_ values using the actin standard curve and these experiments proved the specificity of designed primers and efficacy of the system (Table 3).

**Table 3:** Concentration of the different hordein coding DNA clones

**Hordein primers**	**DNA concentration (amol/μl)**	
	Added DNA*	**Calculated from C**_**t**_**values**
		**Pooled all (15): B1 (5), B3 (4), C (1), D (2), γ1 (2) and γ3 (1)**
**Common B**	5.16	5.71 ± 0.18
**B1**	2.94	2.92 ± 0.11
**B1a**	2.94	2.74 ± 0.55
**JQ867084**	0.63	0.54 ± 0.14
**B3**	2.21	2.41 ± 0.61
**B3a**	2.21	1.81 ± 0.11
**B3b**	0.88	1.09 ± 0.02
**Common C**	0.53	0.51 ± 0.02
**Common D**	0.43	0.51 ± 0.01
**Common γ1**	1.04	0.97 ± 0.07
**Common γ3**	0.54	0.56 ± 0.00
**JQ867080**	0.54	0.47 ± 0.07

*DNA concentration was measured for individual clones and the numbers represent amol/μl DNA in the pool.

Note: the calculation based on three replicates and presented as means ± SD.

B1-hordein clones: JQ859916, JQ859917, JQ867073, JQ867074, JQ867084; B3-hordein clones: JQ867085, JQ867086, JQ867087, JQ867089; C-hordein clone: JQ867090; D-hordein clones: JQ867076, JQ867077; γ1-hordein clones: JQ867078, JQ867079; γ3-hordein clone: JQ867080.

### The stability of reference genes

The genes for *actin, ubiquitin* or *protein translation factor SUI1 homolog (GOS2 protein)* were used as internal references to normalise cDNA concentration between grain samples taken at different developmental stages. The fluctuations of the housekeeping genes were calculated relative to each other during grain development (Table 4). Actin gene showed the most stabile expression, followed by the ubiquitin gene, while the gene for *protein translation factor SUI1 homolog (GOS2 protein)* showed higher expression level at 25DAP but stable expression in the earlier stages (Table 4).

**Table 4:** Stability of selected housekeeping genes in the developing barley grain

**DAP**	**Actin/Ubiquitin**	**Actin/PTF**	**Ubiquitin/Actin**	**Ubiquitin/PTF**	**PTF/Actin**	**PTF/Ubiquitin**
**10**	0.16 ± 0.01	0.12 ± 0.01	6.15 ± 0.19	0.72 ± 0.02	8.56 ± 0.33	1.39 ± 0.05
**15**	0.13 ± 0.03	0.17 ± 0.04	7.53 ± 0.48	1.26 ± 0.08	6.57 ± 0.23	0.79 ± 0.14
**18**	0.14 ± 0.03	0.20 ± 0.04	6.17 ± 0.93	1.42 ± 0.34	5.99 ± 0.24	0.71 ± 0.24
**25**	0.11 ± 0.00	0.08 ± 0.00	9.21 ± 0.32	0.70 ± 0.02	13.19 ± 0.38	1.44 ± 0.04

Ratio: difference in the amount of the two housekeeping genes; calculated as amol x 10^-3^.

### Differential expression of hordein alleles in developing barley (cv. Barke) grain

Quantitative-PCR experiments were performed to; 1) validate the high throughput platform using the same material described in microarray study conducted by Hansen et al. study [16]; 2) extend the gene expression study with our newly designed experimental set up (Figure 1; Table 2). Although three housekeeping genes (*actin, ubiquitin* and *protein translation factor SUI1 homolog)* were used to normalise the data acquired across the development series, we presented the results using actin as the reference gene as this gene proved to be the most stabile during in the studied period (Table 4).

**Figure 4:**
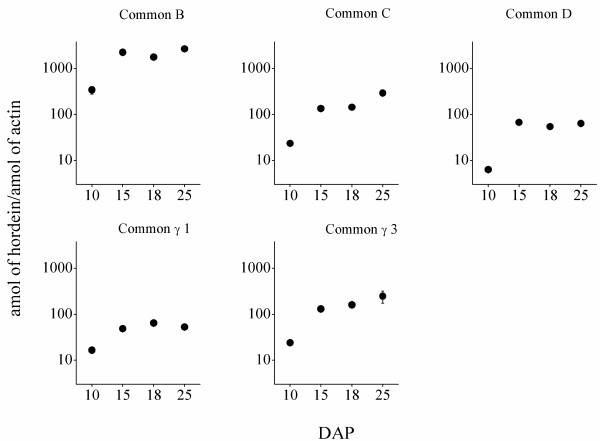
**Expression of the different hordein transcripts in barley cv. Barke grain.** Expression was measured at 10, 15, 18 and 25 days after pollination (DAP). The calculation based on three replicates and presented as means ± SD (some error bars are not visible due to their small size).

The number of moles of transcript corresponding to a specific class of hordein alleles expressed during grain development was calculated using a standard curve created using actin as an internal reference and standard. Our work indicated that the genes encoding hordeins, the major storage proteins of barley, were expressed at different levels. Figure 4 illustrates that throughout development B-hordein transcripts are the most abundant, followed by C-, γ- and D-hordein transcripts.

Further analysis of the temporal expression data shown in Table 5 reveals the percentage contribution of each family to the total amount of hordein transcripts. The total amount of hordein transcripts (100%) was added up from Common B, C, D and γ hordein transcript (amol of hordein/amol of actin) (Additional file 2). The total B-hordein gene expression contribution decreased from 82.86% at 10 DAP to 80.43% at 25 DAP, the total C-hordein gene expression contribution increased from 5.73% to 8.72% over the same period, while D-hordein gene expression levels were 1.55% of the total hordeins at 10 DAP; 2.57% at 15 DAP; 2.48% at 18 DAP and 1.89% at 25 DAP. The γ-hordein group (γ1 and γ3 together) decreased from 9.86 at 10 DAP to 8.96% at 25 DAP (Table 5).

**Table 5:** Proportion of the different hordein groups during grain development using actin for normalisation

**Primer**	**Proportion of hordein group (% of total)**			
	**10 DAP**	**15 DAP**	**18 DAP**	**25 DAP**
**Common B**	82.86	85.44	80.69	80.43
B1	35.02	34.94	29.06	15.84
B1a	12.29	12.20	10.06	7.45
X87232	ND	ND	ND	ND
JQ867084	ND	ND	ND	ND
DQ148297	ND	ND	ND	ND
B2	0.40	0.40	0.40	0.19
B3	35.13	23.54	28.38	33.75
B3a	20.76	15.26	18.64	27.76
B3b	ND	ND	ND	ND
B3c	0.05	0.02	0.02	0.02
DQ826387	0.20	0.14	0.17	0.09
GQ342976	0.19	0.31	0.47	0.99
JQ867088	ND	ND	ND	ND
**Common C**	5.73	5.16	6.59	8.72
C1	0.05	0.08	0.11	0.18
S66938	3.85	4.58	5.08	5.73
**Common D**	1.55	2.57	2.48	1.89
**Common γ1**	4.02	1.86	2.95	1.57
AJ580585	ND	ND	ND	ND
X13508	ND	ND	ND	ND
**Common γ3**	5.84	4.98	7.30	7.39
X72628	ND	ND	ND	ND
JQ867080	ND	ND	ND	ND

*ND*- not detected.

The multiple sequence alignments of the different families coding hordein sequences revealed both polymorphisms with respect to nucleotides but also overall length of the clone and therefore predicted number of amino acids (Table 1). Studying the phylogenetic relationships of the B-hordein groups suggested two major subfamilies (B1 and B3), one minor subfamily (B2) and several subgroups in the major groups but the MW of the transcript and the coded protein had no correlation with the groups (Figure 1; Table 1).

Analysing the percentage contribution of subfamilies and subgroups of subfamilies within the B-hordein family revealed the absolute concentration of B1-group transcripts was high at the beginning of development from 35.02% (10 DAP) to 34.94% (15 DAP) and decreased to 15.84% at 25 DAP. The primer set for the B1a subgroup covers a significant proportion of the B1-subfamily and its contribution to this subfamily was 35.09%, 34.92%, 34.62% and 47.03% for 10, 15, 18 and 25 DAP, respectively. The transcripts from B3-group had a similar expression to B1-group at early grain developmental stages but in contrast to B1-hordeins their expression decreased to about 30% at 15 and 18 DAP and significantly increased and reached again over 41% of the total B-hordein fraction at 25 DAP (Table 5). The B3a subgroup forms a large part of the B3-subfamily and constitutes ~20% of all B-hordeins at early developmental stages and increased to 34.51% at 25 DAP. The increase of the number of transcripts of the B3a subgroup during late development accounts for the change in transcript level for B3-group as a whole.

We found only one representative of the small, newly separated B2-group and its expression was constant and relatively low during grain formation in the studied cultivar Barke (Figure 1; Table 5).

The contribution of the three members of C-hordein to the total C-hordein transcripts was 62.06, 90.31, 78.76 and 67.78% at 10, 15, 18 and 25 DAP respectively (Figure 1; Table 5).

D1-hordeins has 4 representatives while the D2 group contains 1 member. No working primer sets were established to enable the characterisation of the individual members of the two subfamilies as the differences were in the number of the repetitive motifs (Figure 1; Table 5).

### Presence and absence of alleles in cv. Barke

Table 1 illustrates that the sequences sourced from the databases where actually derived from a wide range of cultivars. It was noted that certain primer sets designed to allelic sequences drawn from the database were not able to detect the corresponding sequence in cv. Barke. We were able to test some of these primer sets isolated from cultivar Golden Promise on their own DNA clones. In spite of the fact that the primer sets supported the production of an appropriate PCR product when Golden Promise DNA was used (Table 3) we did not observe a PCR product when tested on the cultivar Barke DNA (Table 5). For example, the allele of the B1-subfamily isolated from cultivar Golden Promise was not expressed in cv. Barke (Table 5). Similarly, we did not obtain a PCR product from the cDNA of cv. Barke when using primer sets designed for the members of B3b subgroup cloned from cv. Golden Promise (JQ867086, JQ867087, JQ867089) (Table 5). Furthermore some of the alleles from other cultivars, for examples B1-subfamily members isolated from cultivars Carlsberg II (X87232) and XQ053 (DQ148297) or γ-sequences from cultivars Carina (X13508), Riso 56 (AJ580585) Carlsberg II (X72628) did not produce a PCR product from Barke cDNA (Table 5). Although DNA representing those alleles was not available for testing we suppose that similar results would be obtained, namely the expression pattern is cultivar dependent.

## Discussion

Genetic sequence alignment is the basis of many evolutionary and comparative studies. When creating a multiple sequence alignment of the collated hordein genes, the number and type of repeats within the genes resulted in gaps in otherwise highly homologous sequences. However when performing phylogenetic analysis of the hordein genes using the multiple sequence alignment of full sequences it became apparent that the algorithm removes the gaps when making evolutionary related comparisons [22,23]. Phylogeny-aware gap placement software has been developed to reduce errors in sequence alignment and evolutionary analysis as Löytynoja and Goldman [24]. However it appears that a typical phylogenetic analysis of sequence data involves five distinct steps, one of which is ‘alignment trimming’ [20] choosing of core areas of genes without extensive repeat supported both the phylogenic analysis and primer design. To study long repetitive regions requires techniques different from those which were described in this study.

The DNA sequence identity of hordein alleles is frequently higher than 95%, therefore designing specific primers for families, subfamilies and individual members is challenging. In the light of the fact the primers set had to be designed to often discriminate single nucleotide changes within alleles we adopted the strategy, which underpins the development of SNP (Single Nucleotide Polymorphism) detection. The SNP detection is based on the ability to discriminate single point mutations and relies on DNA polymerase with proof reading activity, to extend a primer only when its 3'-end is perfectly complementary to the template [25]. Whiley and Sloots [26] used Taqman probes and combination of variously modified primers and they noticed a destabilizing effect for a single base mismatch in the 3'-end while 5'-end is less likely to introduce error. The observation was considered in our experiment and was confirmed in our results: most of the specific primers have a mismatched nucleotide at the 3'-end (Figure 1; Table 2; Additional file 1). In contrast to one fluorescence dye (SYBR® Green I) qPCR assay, Taqman probes can be based either on regular oligonucleotides or on Locked Nucleic Acid (LNA) and detect specific reaction products only [27,28]. However, the method is relatively expensive and a different probe has to be synthesised for each unique target sequence which is a complicated task in the case of large multigene families. In our experiment we proved that SYBR®-Green based detection is sensitive when appropriate primer sets were used and was therefore more economic (Figure 1; Table 2).

Validation of actin standard curve and primer specificity using cloned DNA targets by qRT-PCR reaction showed the robustness of the method. It is recommended that if possible to check the specificity of designed primers with original cloned DNA template but very often this kind of control is beyond the bounds of possibility for a researcher. Furthermore it is advised to design primer pairs were both of primers of the set can distinguish the SNP. It was observed in our experiment as well that the specificity increased when both primers of the set were unique for desired amplicons (Table 2; Additional file 1).

Hansen et al. [16], using microarray derived data, reported variation in the temporal expression of genes coding for barley storage protein family members within the cultivar Barke. The data sets resulting from microarray were validated by using qRT-PCR and the primers were chosen to recognise most members of the same gene family [16]. Using the same field grown material our principle objectives were two fold 1) quantify the total mRNA of specific classes of hordein during development 2) attempt to dissect out the contribution of subclasses and specific alleles within a class during development. We found substantial fluctuation in the contribution of the different families to the total in the different hordein gene fractions; furthermore we were able to distinguish different contribution of the family members to the total hordein gene pool during grain development.

In the case of B-hordein family, it has been suggested that it is a multigene family with approximately 34 members [12]. Previous reports have identified at least three classes of B-hordein on the basis of their cyanogen bromide (CNBr) cleavage patterns and considerable variation was observed in the numbers and amounts of polypeptides of each class present in different genotypes [29]. The work of Kreis et al. [30] verified at least two major subfamilies of B-hordein mRNAs, and thus of genes, associated with the *Hor2* locus. We established similar major subfamilies by the phylogenic analysis of the currently available 29 sequences (Figure 1; Table 1). Most of the available sequences from the databases were not annotated according to groups and just described as B-hordein. Our sequences, recently cloned and submitted to the database (JQ series in Table 1), have the classification presented in this paper. Although we found no correlation between the groups and the MW of the coded proteins we adhered to the classification of B-hordein as described by Shewry [31] who distinguished two groups: B1 (class I and II) and B3 (class III) by MW. Further to the two major groups (B1 and B3) suggested by phylogenetic analysis, one minor group (B2) and several subgroups in the major groups were obtained (Figure 1). The contribution of B1- and B3-subfamilies to the total pool of B-hordein transcripts differs during grain development however it would appear that the B3-subfamily contributes the greater proportion of total B-hordein content in the mature grain (Table 5). Hansen et al. [16] observed a similar tendency for some of B1- and B3-representatives in their microarray analysis. The total of amount C-hordein gene transcripts increased during the developmental period reaching 8.72% of the total, while D-hordein gene expression levels reached the highest level at 15DAP (2.57%). The amount of transcripts for the γ1-hordein subfamily decreased during the studied period while the percentage of γ3 –hordeins appeared to increase throughout development. It was reported by Rechinger et al. [32] that there is role for γ3-hordein in the transport and targeting of prolamin polypeptides so they do not serve only as a storage proteins.

The selection of housekeeping genes is critical for gene expression studies. Actin, GAPDH, tubulin, 18S rRNA and heat shock protein 70 are common reference genes for barley [33-35]. In our experiment we verified two new housekeeping genes: *ubiquitin* and *protein translation factor SUI1 (GOS2 protein) homolog* (Table 1). We checked the stability of these reference genes at different time points and our results implied that the *ubiquitin* and *protein translation factor SUI1 (GOS2 protein) homolog* coding genes (Table 1) can be used for normalisation when gene expression level is studied in grain growth stages and different barley cultivars.

The often neglected fact of the database sequences that they usually originate from different cultivars. It is true for the available hordein sequences as well; they were cloned from many different cultivars. Our result highlighted that presence of some of the alleles are cultivar and/or developmental stage dependent and it should be considered when gene expression studies are performed.

## Conclusions

The method described enabled rapid characterisation of the allelic contribution to the total hordein storage protein transcript population during grain development. Using cheaper SYBR Green labelling in the qRT-PCR reactions was sufficient to distinguish expression levels of large gene families and their members even with high sequence identity. The qRT-PCR validation experiments using cloned DNA targets proved the specificity of designed primers and with the application of the actin standard curve the efficacy of the system was proven as well. We confirmed the stability of the expression of the chosen reference genes during the studied barley grain development period and found substantial fluctuation in the contribution of the different families to the total in the different hordein gene fractions; furthermore we were able to distinguish different contribution of the family members to the total hordein gene pool during grain development. Our result highlighted that presence of some of the alleles are cultivar and/or developmental stage dependent and it should be considered when gene expression studies are performed. The described primer sets could be used as functional marker to help the breeding effort for better storage protein qualities.

## Methods

### Plant material

*Hordeum vulgare* L cv. Barke was grown under field conditions as described Hansen et al. [16]. All the plant material was morphologically and chronologically staged in accordance with internationally recognised criteria of Zadoks code [36]. Individual spikes were tagged at flowering and harvested in the morning (09.00-10.00 h) at 10, 15, 18, and 25 d after pollination (DAP). Developing grains were immediately frozen in liquid nitrogen and stored at −80°C until analysis. Two grains were sampled from the middle of a spike. Three spikes per treatment where sampled and the grains pooled before analysis. Each measurement was repeated three times.

### DNA, RNA isolation and cDNA synthesis

DNA coding individual hordein alleles and actin gene from barley was prepared from plasmid clones using GenElute Plasmid Miniprep kit (Sigma-Aldrich). DNA was measured using DNA Quantitation Kit, Fluorescence Assay (Sigma-Aldrich).

Total RNA was extracted from milled material according to manufacturer’s protocol (FastRNA Pro Green Kit, Bio101 Systems, France). The isolated RNA was treated with DNase according to the manufacturer protocol (Qiagen) to ensure that all genomic DNA was removed. The RNA was re-isolated with FastRNA Pro Green Kit. RNA quality was checked using an Agilent 2001 Bioanalyzer (Agilent Technologies, Inc.). Samples with RNA Integrity Number (RIN) above 7 [37] were used for mRNA extraction with Dynabeads-Oligo (dT)_12–18_ according to manufacturer’s protocol (Invitrogen, Norway). First strand cDNA was prepared using 500 ng (500 ng/μl) of Oligo (dT)_12–18_ primer and Superscript II reverse transcriptase according to manufacturer’s protocol (Invitrogen, USA). The resulting cDNA mixture was diluted to 200 μl by adding 180 μl of MilliQ- H_2_O and stored at −20°C.

### Designing of specific primers for qRT-PCR expression analysis

All available full length gene sequences for hordeins- B, C, D and γ were collected from: EMBL Nucleotide Sequence Database (EMBL), DNA Data Bank of Japan (DDBJ), GenBank at the NCBI (GenBank) and HarvEST database. The accession numbers of the chosen DNA sequences are listed in Table 1. The molecular sequences were aligned using online version of ClustalW2 software [38]. The phylogenetic relationships of the different hordein family members were analysed using the software compiled at Phylogeny.fr using standard module [39,40]. Primer pairs, specific for the whole family, subfamilies of the family and individual sequences were designed manually and their quality was checked by Oligonucleotide primer check software [41]. We were looking for differences between alleles and prioritized primers with mismatched nucleotide at 3′ end. The selected primers are oligonucleotides with a length between 18 and 22 bases and 40-60% of GC content. The amplicon length is between 50 and 150 bp. The selection of *actin, ubiquitin* and *protein translation factor SUI1 homolog (GOS2 protein)* as reference genes was based on the report of Sreenivasulu et al. [42] and the genes were used for normalisation and quantification (Table 1). A list of the primers designed towards the hordein gene families and the reference genes is given in Table 2.

### Quantitative RT-PCR conditions

Quantitative RT-PCR reactions were carried out in triplicate in 384 well microtiter plates (ABI PRISM ^TM^; Applied Biosystems). The total reaction volume was 10 μl which comprised of 5 μl Power SYBR Green Master Mix (Applied Biosystems), 0.5 μM forward and reverse primers (Invitrogen), 1 μl appropriately diluted plasmid DNA, plasmid DNA mix or cDNA. No-template control (NTC) reactions were carried out to check the potential of primer-dimers formation. The qRT- PCR reactions were performed using a 7900HT Sequence Detection System (Applied Biosystems) programmed with the following thermal profile setup: one cycle at 50°C for 2 min; one cycle at 95°C for 10 min; 40 cycles at 95°C for 15 s and 60°C for 1 min. ‘Absolute Quantification’ assay type was used. Data analysis was performed with SDS 2.2.1 software (Applied Biosystems) followed by Microsoft Office Excel 2007 and outliers had been removed.

The DNA standard curve was prepared from a dilution series (10^-1^ to 10^-8^) using DNA isolated from the cloned *actin* gene (HVSMEi0002G07f). The qRT-PCR reactions with primers specific for *actin* gene were performed in triplicate for each concentration. The PCR efficiency was calculated from the slope of the standard curve according to the following formula: E = 10^(−1/slope)^-1 where an efficiency of 1 corresponds to 100% [43]. The C_t_ value obtained for each hordein and reference gene was an average of three PCR reactions on the same cDNA pool. The number of attomoles of individual transcripts was calculated from the weight of specific cDNA derived from the standard curve and corresponding length of coding region (Table 1). The number of moles (amol) of a specific hordein were normalised to the number of moles of housekeeping genes (*actin or ubiquitin* or *protein translation factor SUI1 homolog (GOS2 protein).* The reference genes and standard curve were present on each plate to detect and remove inter-run variation.

We portrayed the hordein content (amol) per actin according to the days after pollination (Figure 4). Due to the wide range of hordein data, we used logarithmic scale for the representation in R 2.15.0 [44].

Our study conforms to the Minimum Information for Publication of Quantitative Real-Time PCR Experiments (MIQE).

### Validation of primer specificity toward target alleles within qRT-PCR reaction

We possess the JQ series (see Table 1) of hordein alleles cloned into plasmid vector. To test the specificity of each primer set which had been designed towards a specific allele or alleles qRT-PCR reaction were carried out on a) the DNA template of the target allele(s) b) non-target allelic DNA templates and c) with pooled samples i.e. all alleles. When the target allele was amplified the amplification plot was visually inspected to verify that the C_t_ values were in optimal range as described previously in the section of qRT-PCR conditions of the M&M. The number of ng amplified by the allele specific primers in the presence of the target template was determined by referencing a standard curve presented in Figure 2. The number of amol was calculated from the ng of DNA derived from the standard curve and corresponding length of coding region (Table 1).

We measured DNA concentrations of 15 hordein clones as described previously in DNA, RNA and cDNA section and carried out the qRT-PCR reactions with selected primers to check validation of the system. The differences between the number of amol estimated from the amplification product and that actually added to the reaction were used to estimate the amplification efficiency (internal control for the quality of the allele specific primer set) (Table 3).

## Abbreviations

AFLPs: Amplified fragment length polymorphisms; cDNA: Complementary DNA; C_t_: Threshold cycle; DAP: Days after pollination; FMs: Functional markers; LNA: Locked nucleic acid; MIQE: Minimum Information for Publication of Quantitative Real-Time PCR Experiments; NTC: No-template control; RFLPs: Restriction fragment length polymorphisms; SNP: Single nucleotide polymorphism; SSRs: Simple sequence repeats; qRT-PCR: Quantitative reverse transcription-polymerase chain reaction; QTINDEL: Quantitative trait insertion – deletion; QTL: Quantitative trait locus; QTN: Quantitative trait nucleotides.

## Competing interests

The authors declare that they have no competing interests.

## Authors’ contributions

AK isolated the RNA, made the cDNAs, designed the primers, carried out the qRT-PCR experiments and evaluated the data. EZ helped with the statistical analysis. SB and EV participated in the planning the experiments, evaluating the data and was involved writing the article together with AK. All authors read and approved the final manuscript.

## Supplementary Material

Additional file 1 **Alignment of B-, C-, D- and γ-hordein sequences found in NCBI databases.** A location of qRT-PCR primers is highlighted. Click here for file

Additional file 2 Abundance of hordein groups during grain development using actin for normalisation. Click here for file

## Author details

^1^Department of Genetics and Biotechnology, Aarhus University, Research Centre Flakkebjerg, Forsøgsvej 1, Slagelse, DK-4200, Denmark. ^2^Verzyme (UK) Ltd., Plas Gogerddan, Aberystwyth, Wales, SY23 3EB, United Kingdom. ^3^MTA-ELTE-MTM Ecology Research Group, Biological Institute, Eötvös Loránd University, Pázmány Péter sétány 1C, Budapest, H-1117, Hungary.
