# Supplementary material for: Quantitative RT-PCR based platform for rapid quantification of the transcripts of highly homologous multigene families and their members during grain development
Source: BMC Plant Biol. 2012 Oct 9;12:184. doi: 10.1186/1471-2229-12-184 (PMC3492166; doi:10.1186/1471-2229-12-184)
Supplement: Additional file 1 — Alignment of B-, C-, D- and γ-hordein sequences found in NCBI databases. A location of qRT-PCR primers is highlighted. [file 1471-2229-12-184-S1.pdf]

B

```
JQ859917        CCACCATTTGGGCTACAACAACCAATTCTATCGCAGCAACAACCATGTACACCACAACAA 60
JQ867075        CCACCATTTGGGCTACAACAACCAATTCTATCGCAGCAACAACCATGTACACCACAACAA 60
JQ867074        CCACCATTTGGGCTACAACAACCAATTCTATCGCAGCAACAACCATGTACACCACAACAA 60
JQ859915        CCACCATTTGGGCTACAACAACCAATTCTATCGCAGCAACAACCATGTACACCACAACAA 60
X53690          CCACCATTTGGGCTACAACAACCAATTCTATCGCAGCAACAACCATGTACACCACAACAA 60
JQ867081        CCACCATTTGGGCTACAACAACGAATTCTATCGCAGCAACAACCATGTACACCACAACAA 60
JQ867083        CCACCATTTGGGCTACAACAACGAATTCTATCGCAGCAACAACCATGTACACCACAACAA 60
JQ867082        CCACCATTTGGGCTACAACAACGAATTCTATCGCAGCAACAACCATGTACACCACAACAA 60
JQ867084        CCACCATTTTGGCAACAACAACCAATTCTATCGCAGCAACAACCATGTACACCACAACAA 60
DQ178602        CCACCATTTGGGCTACAACAACCAATTCTATGGCAGCAACAACCATGTACACCACAACAA 60
DQ267479        CCACCATTTGGGCTACAACAACCAATTCTATGGCAGCAACAACCATGTACACCACAACAA 60
JQ867073        CCACCATTTGGGCTACAACAACCAATTCTATCGCAGCAACAACCATGTACACCACAACAA 60
                         X87232_FW--->
X87232          CCACCATTTGGGCTACAACAACCAATTCTGTCGCAACAACAACCATGTACACCACAACCA 60
JQ859916        CCACCATTTGGGCTACAACAACCAATTCTATCGCAACAACAACCATGTACACCACAACAA 60
DQ148297        CCACCATTTGGGCTACAACAACCGATTCTATCGCAACAACAACCATGTACACCACAACAA 60
X03103          CCACCATTTGGGCTACAACAACCAATTCTATCGCAGCAACAACCATGTACACCACAACAA 60
JQ867086        CCACGATTTTGGCAACAACAACCAGTTCTATCGCAGCAACAACCATGTACACAAGAACAA 60
JQ867087        CCACCATTTTGGCAACAACAACCAGTTCTATCGCAGCAACAACCATGTACACAAGAACAA 60
JQ867089        CCACCATTTTGGCAACAACAACCAGTTCTATCGCAGCAACAACCATGTACACAAGAACAA 60
JQ867085        CCACCATTTTGGCAACAACAACCAGTTCTATCGCAGCAACAACCATGTACACAAGAACAA 60
GQ342970        CCACCATTTTGGCAACAACAACCAGTTCTATCGCAGCAACAACCATGTACACAAGACCAA 60
GQ342971        CCACCATTTTGGCAACAACAACCAGTTCTATCGCAGCAACAACCATGTACACAAGACCAA 60
DQ267478        CCACCATTTTGGCAACAACAACCAGTTCTATCGCAGCAACAACCATGTACACAAGACCAA 60
GQ342972        CCACCATTTTGGCAACAACAACCAGTTCTATCGCAGCAACAACCATGTACACAAGACCAA 60
GQ342976        CCACCATTTTGGCAACAACAACCAGTTCTATCGCAGCAACAACCATGTACACAAGACCAA 60
GQ342973        CCACCATTTTGGCAACAACAACCAGTTCTATCGCAGCAACAACCATGTACACAAGACCAA 60
GQ342975        CCACCATTTTGGCAACAACAACCAATTCTATCGAAGCAACAACCATGTACACCACAACAA 60
JQ867088        CCACCATTTTGGCAACAACAACCAGTTCTATCGCAGCAACAACCATGTACACCACAACAA 60
DQ826387        CCACCATTTTGGCAACAACAACCAGTTCTATCGCAGCAACAACCATGTACACAAGACCAA 60
                            * ********   **** * * * **************** * * * *

JQ859917        ACACCACTCCCACAAGGACAACTGTATCAAACGCTTCTGCAACTACAAATACCCTATGTT 120
JQ867075        ACACCACTCCCACAAGGACAACTGTATCAAACGCTTCTGCAACTACAAATACCCTATGTT 120
JQ867074        ACACCACTCCCACAAGGACAACTGTATCAAACGCTTCTGCAACTACAAATACCCTATGTT 120
JQ859915        ACACCACTCCCACAAGGACAACTGTATCAAACGCTTCTGCAACTACAAATACCCTATGTT 120
X53690          ACACCACTCCCACAAGGACAACTGTATCAAACGCTTCTGCAACTACAAATACCCTATGTT 120
JQ867081        ACACCACTCCCACAAGGACAACTGTATCAAACGCTTCTGCAACTACAAATACCCTATGTT 120
JQ867083        ACACCACTCCCACAAGGACAACTGTATCAAACGCTTCTGCAACTACAAATACCCTATGTT 120
JQ867082        ACACCACTCCCACAAGGACAACTGTATCAAACGCTTCTGCAACTACAAATACCCTATGTT 120
JQ867084        ACACCACTCCCACAAGGACAACAAGATCAAATGCTTGTGCAAGTACAAATACCATTTGTT 120
DQ178602        ACACCACTCCCACAAGGACAACTGTATCAAACGCTTCTGCAACTACAAATACCCTATGTT 120
DQ267479        ACACCACTCCCACAAGGACAACTGTATCAAACGCTTCTGCAACTACAAATACCCTATGTT 120
JQ867073        ACACCACTCCCACAAGGACAACTGTATCAAACGCTTCTGCAACTACAAATACCCTATGTT 120
                 <---- X87232_RV
X87232          ACACCACTCCCACAAGGACAACTGTACCAAACGCTTCTGCAACTACAAATACCCTATGTT 120
JQ859916        ACACCACTCCCACAAGGACAACTGTACCAAACGCTTCTGCAACTACAAATACCCTATGTT 120
DQ148297        ACACCACTCCCACAAGGACAACTGTACCAAACGCTTCTGCAACTACAAATACCCAATGTT 120
X03103          ACACCACTCCCACAAGGACAACTGTACCAAACGCTTCTGCAACTACAAATACAATATGTT 120
JQ867086        ACACCACTCCTACAAGAACAACAAGATCAAATGCTTCTGCAAGTACAAATACCATTTGTT 120
JQ867087        ACACCACTCCTACAAGAACAACAAGATCAAATGCTTCTGCAAGTACAAATACCATTTGTT 120
JQ867089        ACACCACTCCTACAAGAACAACAAGATCAAATGCTTGTGCAAGTACAAATACCATTTGTT 120
JQ867085        ACACCACTCCTACAAGAACAACAAGATCAAATGCTTCTGCAAGTACAAATACCATTTGTT 120
GQ342970        ACACCACTCCTACAAGAACAACAAGATCAAATGCTTCTGCAAGTACAAATACCATTTGTT 120
GQ342971        ACACCACTCCTACAAGAACAACAAGATCAAATGCTTCTGCAAGTACAAATACCATTTGTT 120
DQ267478        ACACCACTCCTACAAGAACAACAAGATCAAATGCTTCTACAAGTACAAATACCATTTGTT 120
GQ342972        ACACCACTCCTACAAGAACAACAAGATCAAATGCTTCTACAAGTACAAATACCATTTGTT 120
                      GQ342976_FW--->
GQ342976        ACACCACTCCTACAAGAACAACAAGACCAAATGCTTCTACAAGTACAAATACCATTTGTT 120
GQ342973        ACACCACTCCTACAAGAACAACAAGATCAAATGCTTCTACAAGTACAAATACCATTTGTT 120
GQ342975        CCACCACTCCCACAAGGACAACAAGATCAAATGCTTGTGCAAGTACAAATACCATTTGTC 120
JQ867088        ACACCACTCCCACAAGGACAACAAGATCAAATGCTTGTGCAAGTACAAATACCATTTGTT 120
DQ826387        ACACCACTCCTACAAGAACAACAAGATCAAATGCTTCTGCAAGTACAAATACCATTTGTT 120
                 ********* ***** *****   * **** **** * *** *********    ***

JQ859917        CATCCATCTATATTGCAACAGCTAAACCCATGCAAGGTATTCCTCCAGCAGCAGTGCAGC 180
JQ867075        CATCCATCTATATTGCAACAGCTAAACCCATGCAAGGTATTCCTCCAGCAGCAGTGCAGC 180
```

```
JQ867074        CATCCATCTATATTGCAACAGCTAAACCCATGCAAGGTATTCCTCCAGCAGCAGTGCAGC 180
JQ859915        CATCCATCTATATTGCAACAGCTAAACCCATGCAAGGTATTCCTCCAGCAGCAGTGCAGC 180
X53690          CATCCATCTATATTGCAACAGCTAAACCCATGCAAGGTATTCCTCCAGCAGCAGTGCAGC 180
JQ867081        CATCCATCTATTTTGCAACAGCTAAACCCATGCAAGGTATTCCTCCAGCAGCAGTGCAGC 180
JQ867083        CATCCATCTATTTTGCAACAGCTAAACCCATGCAAGGTATTCCTCCAGCAGCAGTGCAGC 180
JQ867082        CATCCATCTATTTTGCAACAGCTAAACCCATGCAAGGTATTCCTCCAGCAGCAGTGCAGC 180
JQ867084        CATCCATCTATTTTGCAACAGCTAAACCCATGCAAGGTATTCCTCCAGCAGCAGTGCAGC 180
DQ178602        CATCCTTCTATTTTGCAACAACTAAACCCATGCAAGGTATTCCTCCAGCAGCAGTGCAAC 180
DQ267479        CATCCTTCTATTTTGCAACAACTAAACCCATGCAAGGTATTCCTCCAGCAGCAGTGCAAC 180
JQ867073        CATCCATCTATATTGCAACAGCTAAACCCATGCAAGGTATTCCTCCAGCAGCAGTGCAGC 180
X87232          CAACCATCTATTTTGCAACAGCTAACCCCATGCAAGGTATTCCTCCAGCAGCAGTGCAGC 180
JQ859916        CAACCATCTATTTTGCAACAGCTAAACCCATGCAAGGTATTCCTCCAGCAGCAGTGCAGC 180
DQ148297        CAACCATCTATTTTGCAACAGCTAAACCCATGCAAGGTATTCCTCCAGCAGCAGTGCAGC 180
X03103          CATCCATCTATTTTGCAACAGCTAAACCCATGCAAGGTATTCCTCCAGCAGCAGTGCAGC 180
JQ867086        CATCCATCTATTTTGCAGCAGCTAAACCCATGCAAGGTATTCCTCCAGCAGCAGTGCAGC 180
JQ867087        CATCCATCTATTTTGCAGCAGCTAAACCCATGCAAGGTATTCCTCCAGCAGCAGTGCAGC 180
JQ867089        CATCCATCTATTTTGCAGCAGCTAAACCCATGCAAGGTATTCCTCCAGCAGCAGTGCAGC 180
JQ867085        CATCCATCTATTTTGCAGCAGCTAAACCCATGCAAGGTATTCCTCCAGCAGCAGTGCAGC 180
GQ342970        CATCCATCTATTTTGCAGCAGCTAAACCCATGCAAGGTATTCCTCCAGCAGCAGTGCAGC 180
GQ342971        CATCCATCTATTTTGCAGCAGCTAAACCCATGCAAGGTATTCCTCCAGCAGCAGTGCAGC 180
DQ267478        CATCCATCTATTTTGCAGCAGCTAAACCCATGCAAGGTATTCCTCCAGCAGCAGTGCAGC 180
GQ342972        CATCCATCTATTTTGCAGCAGCTAAACCCATGCAAGGTATTCCTCCAGCAGCAGTGCAGC 180
                                   <---- GQ342976_RV
GQ342976        CATCCATCTATTTTGCAGCAGCTAAACCCATGCAAGGTATTCCTCCAGCAGCAGTGCAGC 180
GQ342973        CATCCATCTATTTTGCAGCAGCTAAACCCATGCAAGGTATTCCTCCAGCAGCAGTGCAGC 180
GQ342975        CATCCATCTATTTTGCAGCAGCTAAACCCATGCAAGGTATTCCTCCAGCAGCAGTGCAGC 180
JQ867088        CATCCATCTATTTTGCAGCAGCTAAACCCATGCAAGGTATTCCTCCAGCAGCAGTGCAGC 180
DQ826387        CATCCATCTATTTTGCAGCAGCTAAACCCATGCAAGGTATTCCTCCAGCAGCAGTGCAGC 180
                ** ** ***** ***** ** **** ******************************** *

JQ859917        CCCGTGCGAATGCCACAACTTATTGCTAGGTTGCAAATGTTGCAGCAGAGCAGTTGCCAT 240
JQ867075        CCCGTGCGAATGCCACAACTTATTGCTAGGTTGCAAATGTTGCAGCAGAGCAGTTGCCAT 240
JQ867074        CCCGTGCGAATGCCACAACTTATTGCTAGGTTGCAAATGTTGCAGCAGAGCAGTTGCCAT 240
JQ859915        CCCGTGCGAATGCCACAACTTATTGCTAGGTTGCAAATGTTGCAGCAGAGCAGTTGCCAT 240
X53690          CCCGTGCGAATGCCACAACTTATTGCTAGGTTGCAAATGTTGCAGCAGAGCAGTTGCCAT 240
JQ867081        CCCGTGCGAATGCCACAACTTATTGCTAGGTTGCAAATGTTGCAGCTGAGCAGTTGCCAT 240
JQ867083        CCCGTGCGAATGCCACAACTTATTGCTAGGTTGCAAATGTTGCAGCTGAGCAGTTGCCAT 240
JQ867082        CCCGTGCGAATGCCACAACTTATTGCTAGGTTGCAAATGTTGCAGCTGAGCAGTTGCCAT 240
JQ867084        CCCGTGCGAATGCCACAACTTATTGCTAGGTTGCAAATGTTGCAGCTGAGCAGTTGCCAT 240
DQ178602        CCCGTGCGAATGCCACAACTTATTGCTAGGTTGCAAATGTTGCAGCAGAGCAGTTGCCAT 240
DQ267479        CCCGTGCGAATGCCACAACTTATTGCTAGGTTGCAAATGTTGCAGCAGAGCAGTTGCCAT 240
JQ867073        CCCGTGCGAATGCCACAACTTATTGCTAGGTTGCAAATGTTGCAGCAGAGCAGTTGCCAT 240
X87232          CCCGTGCGAATGCCACAACTTATTGCTAGGTCGCAAATGTTGCAGCAGAGCAGTTGCCAT 240
JQ859916        CCCGTGCGAATGCCACAACTTATTGCTAGGTCGCAAATGTTGCAGCAGAGCAGTTGCCAT 240
DQ148297        CCCGTGCGAATGCAACAACTTATTGCTAGGTCGCAAATGTTTCAGCAGAGCAGTTGCCAT 240
X03103          CCTGTGCCAGTGCCACAACGTATTGCTAGGTCGCAAATGTTGCAGCAGAGCAGTTGCCAT 240
                             B3b_FW ---->
JQ867086        CCTGTGGCAATGTCACAACGTATTGCAAGGTCGCAGATGTTGCAACAGAGCAGTTGCCAT 240
JQ867087        CCTGTGGCAATGTCACAACGTATTGCAAGGTCGCAGATGTTGCAACAGAGCAGTTGCCAT 240
JQ867089        CCTGTGGCAATGTCACAACGTATTGCAAGGTCGCAGATGTTGCAACAGAGCAGTTGCCAT 240
JQ867085        CCTGTGGCAATGTCACAACGTATTGCAAGGTCGCAAATGTTGCAGCAGAGCAGTTGCTAT 240
GQ342970        CCTGTGGCAATGTCACAACGTATTGCAAGGTCGCAAATGTTGCAGCAGAGCAGTTGCTAT 240
GQ342971        CCTGTGGCAATGTCACAACGTATTGCAAGGTCGCAAATGTTGCAGCAGAGCAGTTGCTAT 240
DQ267478        CCTGTGGCAATGTCTCAACGTATTGCAAGGTCGCAAATGTTGCAACAGAGCAGTTGCCAT 240
GQ342972        CCTGTGGCAATGTCTCAACGTATTGCAAGGTCGCAAATGTTGCAACAGAGCAGTTGCCAT 240
GQ342976        CCTGTGGCAATGTCTCAACGTATTGCAAGGTCGCAAATGTTGCAACAGAGCAGTTGCCAT 240
GQ342973        CCTGTGGCAATGTCACAACGTATTGCAAGGTCACAAATGTTGCAGCAGAGCAGTTGCCAT 240
GQ342975        CCTGTGGCAATGTCACAACGTATTGCAAGGTCACAAATGTTGCAGCAGAGCAGTTGCCAT 240
JQ867088        CCTGTGGCAATGTCACAACGTATTGCAAGGTCGCAAATGTTGCAGCAGAGCAGTTGCTAT 240
DQ826387        CCTGTGGCAATGTCTCAACGTATTGCAAGGTCGCAAATGTTGCAACAGAGCAGTTGCCAT 240
                ** ***  * **   **** ****** ****  ** ***** ** * ********** **
                                  B1a_FW ---->
JQ859917        GTGTTGCAGCAACAATGTTGCCAGCAACTGCCGCAAATCTCCGAACAATTCCGCCATGAG 300
JQ867075        GTGTTGCAGCAACAATGTTGCCAGCAACTGCCGCAAATCTCCGAACAATTCCGCCATGAG 300
JQ867074        GTGTTGCAGCAACAATGTTGCCAGCAACTGCCGCAAATCTCCGAACAATTCCGCCATGAG 300
JQ859915        GTGTTGCAGCAACAATGTTGCCAGCAACTGCCGCAAATCTCCGAACAATTCCGCCATGAG 300
X53690          GTGTTGCAGCAACAATGTTGCCAGCAACTGCCGCAAATCTCCGAACAATTCCGCCATGAG 300
JQ867081        GTGTTGCAGCAACAATGTTGCCAGCAACTGCCGCAAATCTCCGAACAATTCCGCCATGAG 300
JQ867083        GTGTTGCAGCAACAATGTTGCCAGCAACTGCCGCAAATCTCCGAACAATTCCGCCATGAG 300
JQ867082        GTGTTGCAGCAACAATGTTGCCAGCAACTGCCGCAAATCTCCGAACAATTCCGCCATGAG 300
```

```
JQ867084        GTGTTGCAGCAACAATGTTGCCAGCAACTGCCGCAAATCTCCGAACAATTCCGCCATGAG 300
DQ178602        GTGTTGCAGCGACAATGTTGCCAGCAACTGCCGCAAATCTCCGAACAATTCCGCCATGAG 300
DQ267479        GTGTTGCAGCGACAATGTTGCCAGCAACTGCCGCAAATCTCCGAACAATTCCGCCATGAG 300
JQ867073        GTGTTGCAGCAACAATGTTGCCAGCAACTGCCGCAAATCTCCGAACAATTCCGCCATGAG 300
X87232          GTGTTGCAGCAACAATGTTGCCAGCAACTGCCGCAAATCCCCGAACAATTCCGCCATGAG 300
JQ859916        GTGTTGCAGCAACAATGTTGCCAGCAACTGCCGCAAATCCCCGAACAATTCCGCCATGAG 300
DQ148297        GTGTTGCAGCAACAATGTTGCCAGCAACTGCCGCAAATCCCCGAACAATTCCGCCATGAG 300
X03103          GTGTTGCAGCAACAATGTTGCCAGCAACTACCCCAAATCCCCGAACAATTCCGTCATGAG 300
                                  <---- B3b_RV
JQ867086        GTGTTGCAGCAACAGTGTTGCCAACAACTGCCGCAAATCCCCGAACAACTCCGCCATGAG 300
JQ867087        GTGTTGCAGCAACAGTGTTGCCAACAACTGCCGCAAATCCCCGAACAACTCCGCCATGAG 300
JQ867089        GTGTTGCAGCAACAGTGTTGCCAACAACTGCCGCAAATTCCCGAACAACTCCGCCATGAG 300
JQ867085        GTGTTGCAGCAACAATGTTGCCAACAACTGCCGCAAATCCCCGAACAATTCCGCCATGAG 300
GQ342970        GTGTTGCAGCAACAATGTTGCCAACAACTGCCGCAAATCCCCGAACAAATCCGCCATGAG 300
GQ342971        GTGTTGCAGCAACAATGTTGCCAACAACTGCCGCAAATCCCCGAACAAATCCGCCATGAG 300
DQ267478        GTGTTGCAGCAACAATGTTGCCAACAACTGCCGCAAATCCCCGAACAAATCCGCCATGAG 300
GQ342972        GTGTTGCAGCAACAATGTTGCCAACAACTGCCGCAAATCCCCGAACAAATCCGCCATGAG 300
GQ342976        GTGTTGCAGCAACAATGTTGCCAACAACTGCCGCAAATCCCCGAACAAATCCGCCATGAG 300
GQ342973        GTGTTGCAGCAACAATGTTGCCAACAACTGCCGCAAATCCCCGAACAATTCCGCCATGAG 300
GQ342975        GTGTTGCAGCAACAATGTTGCCAACAACTGCCGCAAATCCCCGAACAATTCCGCCATGAG 300
JQ867088        GTGTTGCAGCAACAATGTTGCCAACAACTGCCGCAAATCCCCGAACAATTCCGCCATGAG 300
DQ826387        GTGTTGCAGCAACAATGTTGCCAACAACTGCCGCAAATCCCCGAACAAATCCGCCATGAG 300
                ********** *** ******** ***** ** *****  ******** **** ******

                                                B1_FW---->
JQ859917        GCAATCCGTGCAATCGTCTACTCTATCTTTCTGCAAGAACAACCCCAACAGTCGGTCCAA 360
JQ867075        GCAATCCGTGCAATCGTCTACTCTATCTTTCTGCAAGAACAACCCCAACAGTCGGTCCAA 360
JQ867074        GCAATCCGTGCAATCGTCTACTCTATCTTTCTGCAAGAACAACCCCAACAGTCGGTCCAA 360
JQ859915        GCAATCCGTGCAATCGTCTACTCTATCTTTCTGCAAGAACAACCCCAACAGTCGGTCCAA 360
X53690          GCAATCCGTGCAATCGTCTACTCTATCTTTCTGCAAGAACAACCCCAACAGTCGGTCCAA 360
JQ867081        GCAATCCGTGCAATCGTCTACTCTATCTTTCTGCAAGAACAACCCCAACAGTCGGTCCAA 360
JQ867083        GCAATCCGTGCAATCGTCTACTCTATCTTTCTGCAAGAACAACCCCAACAGTCGGTCCAA 360
JQ867082        GCAATCCGTGCAATCGTCTACTCTATCTTTCTGCAAGAACAACCCCAACAGTCGGTCCAA 360
                                                          JQ867084_FW---->
JQ867084        GCAATCCGTGCAATCGTCTACTCTATCTTTCTGCAAGAACAACCCCAACAGTCGGTCCAA 360
DQ178602        GCAATCCGTGCAATCGTCTACTCTATCTTTCTGCAAGAACAACCCCAACAGTCGGTCCAA 360
DQ267479        GCAATCCGTGCAATCGTCTACTCTATCTTTCTGCAAGAACAACCCCAACAGTCGGTCCAA 360
JQ867073        GCAATCCGTGCAATCGTCTACTCTATCTTTCTGCAAGAACAACCCCAACAGTCGGTCCAA 360
X87232          GCAATCCGTGCAATCGTCTACTCTATCTTTCTGCAAGAACAACCCCAACAGTCGGTCCAA 360
JQ859916        GCAATCCGTGCAATCGTCTACTCTATCTTTCTGCAAGAACAACCCCAACAGTCGGTCCAA 360
DQ148297        GCAATCCGTGCAATCGTCTACTCTATCTTTCTGCAAGAACAACCCCAACAGTCGGTCCAA 360
X03103          GCAATCCGTGCAATCGTCTATTCTATCTTCCTGCAAGAACAACCCCAACAGTTGGTCGAA 360
                      B3_FW---->    B3a_FW---->
JQ867086        GCAGTCCGTGCAATCGTCTACTCTATCGTCCTGCAAGAACAATCCCTACAATTGGTCCAA 360
JQ867087        GCAGTCCGTGCAATCGTCTACTCTATCGTCCTGCGAGAACAATCCCTACAATTGGTCCAA 360
JQ867089        GCAGTCCGTGCAATCTTCTACTCTATCGTCCTGCAAGAACAATCCCTACAATTGGTCCAA 360
JQ867085        GCAGTCCGTGCAATCGTCTACTCTATCGTCCTGCAAGAACAATCCCTACAATTGGTCCAA 360
GQ342970        GCAGTCCGTGCAATCGTCTACTCTATCGTCCTGCAAGAACAACCCCTACAATTGGTCCAA 360
GQ342971        GCAGTCCGTGCAATCGTCTACTCTATCGTCCTGCAAGAACAACCCCTACAATTGGTCCAA 360
DQ267478        GCAGTCCGTGCAATCGTCTACTCTATCGTCCTGCAAGAACAACCCCTACAATTGGTCCAA 360
GQ342972        GCAGTCCGTGCAATCGTCTACTCTATCGTCCTGCAAGAACAACCCCTACAATTGGTCCAA 360
GQ342976        GCAGTCCGTGCAATCGTCTACTCTATCGTCCTGCAAGAACAACCCCTACAATTGGTCCAA 360
GQ342973        GCAGTCCGTGCAATCGTCTACTCTATCGTCCTGCAAGAACAACCCCAACAATTGGTCCAA 360
GQ342975        GCAGTCCGTGCAATCGTCTACTCTATCGTCCTGCAAGAACAACCCCAACAATTGGTCCAA 360
JQ867088        GCAGTCCGTGCAATCGTCTACTCTATCGTCCTGCAAGAACAACCCCAACAATTGGTCCAA 360
DQ826387        GCAGTCCGTGCAATCGTCTACTCTATCGTCCTGCAAGAACAACCCCTACAATTGGTCCAA 360
                *** *********** **** ****** * **** ******* *** *** * **** **
                              <---- B1a_RV                          Common B_FW---->
JQ859917        GGTGTATCCCAAACCCAACAACAGTTGCAGCAGGAGCAAGTTGGACAATGTTCTTTCCAA 420
JQ867075        GGTGTATCCCAAACCCAACAACAGTTGCAGCAGGAGCAAGTTGGACAATGTTCTTTCCAA 420
JQ867074        GGTGTATCCCAAACCCAACAACAGTTGCAGCAGGAGCAAGTTGGACAATGTTCTTTCCAA 420
JQ859915        GGTGTATCCCAAACCCAACAACAGTTGCAGCAGGAGCAAGTTGGACAATGTTCTTTCCAA 420
X53690          GGTGTATCCCAAACCCAACAACAGTTGCAGCAGGAGCAAGTTGGACAATGTTCTTTCCAA 420
JQ867081        GGTGTATCCCAAACCCAACAACAGTTGCAGCAGGAGAAAGTTGGACAATGTTCTTTCCAA 420
JQ867083        GGTGTATCCCAAACCCAACAACAGTTGCAGCAGGAGAAAGTTGGACAATGTTCTTTCCAA 420
JQ867082        GGTGTATCCCAAACCCAACAACAGTTGCAGCAGGAGAAAGTTGGACAATGTTCTTTCCAA 420
                                                <---- JQ867084_RV
JQ867084        GGAGTATCCCAAACCCAACAACAGTTGCAGCAGGAGAAAGTTGGACAATGTTCTTTCCAA 420
DQ178602        GGTGTATCCCAACCCCAAAAACAGTTGCAGCAGGAGCAAGTTGGACAATGTTCTTTCCAA 420
```

```
DQ267479        GGTGTATCCCAACCCCAAAAACAGTTGCAGCAGGAGCAAGTTGGACAATGTTCTTTCCAA 420
JQ867073        GGTGTATCCCAAACCCAACAACAGTTGCAGCAGGAGCAAGTTGGACAATGTTATTTCCAA 420
X87232          GGTGCCTCCCAACCCCAACAACAGTTGCAGGAGGAGCAAGTCGGACAATGTTATTTCCAA 420
JQ859916        GGTGTCTCCCAACCCCAACAACAGTTGCAGCAGGAGCAAGTCGGACAATGTTATTTCCAA 420
DQ148297        GGTGTCTCCCAACCCCAACAACAGTTGCAACAGGAGCAAGTCGGACAATGTTATTTCCAA 420
                                              B2_FW
X03103          GGTGTCTCCCAACCACAACAACAGTTGTGGCCGCAGCAAGTCGGACAATGTTCTTTCCAA 420
                   B3_RV                        B3a_RV
JQ867086        GGTGTCTCCCAACCCCAACAACAGTCACAACAGCAACAAGTCGGACAATGTTCTTTCCAA 420
JQ867087        GGTGTCTCCCAACCCCAACAACAGTCACAACAGCAACAAGTCGGACAATGTTCTTTCCAA 420
JQ867089        GGTGTCTCCCAACCCCAACAACAGTCACAACAGCAACAAGTCGGACAATGTTCTTTCCAA 420
JQ867085        GGTGTCTCCCAACCCCAACAACAGTCACAACAGCAACAAGTCGGACAATGTTCTTTCCAA 420
GQ342970        GGTGTCTCCCAACCCCAACAACAGTCACAACAGCAACAAGTCGGACAATGTTCTTTCCAA 420
GQ342971        GGTGTCTCCCAACCCCAACAACAGTCACAACAGCAACAAGTCGGACAATGTTCTTTCCAA 420
DQ267478        GGTGTCTCCCAACCCCAACAACAGTCACAACAGCAACAAGTCGGACAATGTTCTTTCCAA 420
GQ342972        GGTGTCTCCCAACCCCAACAACAGTCACAACAGCAACAAGTCGGACAATGTTCTTTCCAA 420
GQ342976        GGTGTCTCCCAACCCCAACAACAGTCACAACAGCAACAAGTCGGACAATGTTCTTTCCAA 420
GQ342973        GGTATCTCCCAACCCCAACAACAGTCGCAGCTACAGCAAGTCGGACAATGTTCTTTCCAA 420
GQ342975        GGTGTCTCCCAACCCCAACAACAGTCGCAGCTACAGCAAGTCGGACAATGTTCTTTCCAA 420
                                JQ867088_FW
JQ867088        GGTGTCTCCCAACCCCAACAACAGTCGCAGCTACATCAAGTCGGACAATGTTCTTTCCAA 420
DQ826387        GGTGTCTCCCAACCCCAAAAACAGTTGGGGCAGCAGCAAGTCGGACAATGTTCTTTCCAA 420
                **    ****** * *** ******         *  **** ********** *******


JQ859917        CAACCTCAACCACAACAACTTGGTCAAGCACAACAGGTACCACAAAGTGTTTTCTTGCAG 480
JQ867075        CAACCTCAACCACAACAACTTGGTCAAGCACAACAGGTACCACAAAGTGTTTTCTTGCAG 480
JQ867074        CAACCTCAACCACAACAACTTGGTCAAGCACAACAGGTACCACAAAGTGTTTTCTTGCAG 480
JQ859915        CAACCTCAACCACAACAACTTGGTCAAGCACAACAGGTACCACAAAGTGTTTTCTTGCAG 480
X53690          CAACCTCAACCACAACAGCTTGGTCAAGCACAACAGGTACCACAAAGTGTTTTCTTGCAG 480
JQ867081        CAACCTCAACCACAACAACTTGGTCAACCACAGCAGGTACCACAAAGTGTTTTCTTGCAG 480
JQ867083        CAACCTCAACCACAACAACTTGGTCAACCACAGCAGGTACCACAAAGTGTTTTCTTGCAG 480
JQ867082        CAACCTCAACCACAACAACTTGGTCAACCACAGCAGGTACCACAAAGTGTTTTCTTGCAG 480
JQ867084        CAACCTCAACCACAACAACTTGGTCAACCACAGCAGGTACCACAAAGTGTTTTCTTGCAG 480
DQ178602        CAACCTCAACCACAACAACTTGGTCAACCACAGCAGGTACCACACAGTGTTTTCTTGCAG 480
DQ267479        CAACCTCAACCACAACAACTTGGTCAACCACAGCAGGTACCACACAGTGTTTTCTTGCAG 480
JQ867073        CAACCTCAACCACAACAACTTGGTCAACCACAACAGGTACCACAGAGTGTTTTCTTGCAG 480
X87232          CAACCTCAACCACAACAACTTGGTCAACCACAACAGGTACCACAGAGTGTTTTCTTGCAG 480
JQ859916        CAACCTCAACCACAACAACTTGGTCAACCACAACAGGTACCACAGAGTGTTTTCTTGCAG 480
DQ148297        CAACCTCAACCACAACAG---GGTCAACAACAGCAAGTGCCACAGAGTGTTTTCTTGCAG 477
                                              B2_RV
X03103          CAACCTCAACCACAACAAGTTGGTCAACAACAACAGGTACCGCAGAGTGCTTTCTTGCAG 480
JQ867086        CAACCTCAACCACAACAGG---GTCAACAACAGCAAGTGCCACAGAGTGTTCTCTTGCAG 477
JQ867087        CAACCTCAACCACAACAGG---GTCAACAACAGCAAGTGCCACAGAGTGTTCTCTTGCAG 477
JQ867089        CAACCTCAACCACAACAGG---GTCAACAACAGCAAGTGCCACAGAGTGTTCTCTTGCAG 477
JQ867085        CAACCTCAACCACAGCAGG---GTCAACAACAGCAAGTGCCACAGAGTGTTCTCTTGCAG 477
GQ342970        CAACCTCAACCACAACAGG---GTCAACAACAGCAAGTGCCACAGAGTGTTTTCTTGCAG 477
GQ342971        CAACCTCAACCACAACAGG---GTCAACAACAGCAAGTGCCACAGAGTGTTTTCTTGCAG 477
DQ267478        CAACCTCAACCACAACAGG---GTCAACAACAGCAAGTGCCACAGAGTGTTTTCTTGCAG 477
GQ342972        CAACCTCAACCACAACAGG---GTCAACAACAGCAAGTGCCACAGAGTGTTTTCTTGCAG 477
GQ342976        CAACCTCAACCACAACAGG---GTCAACAACAGCAAGTGCCACAGAGTGTTTTCTTGCAG 477
                                  B3c_FW
GQ342973        CAACCTCAACCACAACAGG---GTAAACAACAGCAAGTGCCACATAGTGTTTTCTTGCAG 477
GQ342975        CAACCTCAACCACAACAGG---GTAAACAACAGCAAGTGCCACATAGTGTTTTCTTGCAG 477
JQ867088        CAACCTCAACCACAACAGG---GTCAACAACAGCAAGTGCCACAGAGTGTTTTCTTGCAG 477
                   DQ826387_FW
DQ826387        CAACCTCAACCACAACAAGTTGGCCAACAACAACAGGTACCACAGAGTGCTTTCTTGCAG 480
                ************** **      *  **   *** ** ** ** ** **** * ********
                                                  Common B_RV/B1_RV
JQ859917        CCACACCAGATAGCTCAGCTTGAGGCGACGACTTCCATTGCGCTGCGTACCCTACCAAGG 540
JQ867075        CCACACCAGATAGCTCAGCTTGAGGCGACGACTTCCATTGCGCTGCGTACCCTACCAAGG 540
JQ867074        CCACACCAGATAGCTCAGCTTGAGGCGACGACTTCCATTGCGCTGCGTACCCTACCAAGG 540
JQ859915        CCACACCAGATAGCTCAGCTTGAGGCGACGACTTCCATTGCGCTGCGTACCCTACCAAGG 540
X53690          CCACACCAGATAGCTCAGCTTGAGGCGACGACTTCCATTGCGCTGCGTACCCTACCAAGG 540
JQ867081        CCACACCAGATAGCTCAGCTTGAGGCGACGACTTCCATTGCGCTGCGTACCCTACCAAGG 540
JQ867083        CCACACCAGATAGCTCAGCTTGAGGCGACGACTTCCATTGCGCTGCGTACCCTACCAAGG 540
JQ867082        CCACACCAGATAGCTCAGCTTGAGGCGACGACTTCCATTGCGCTGCGTACCCTACCAAGG 540
JQ867084        CCACACCAGATAGCTCAGCTTGAGGCGACGACTTCCATTGCGCTGCGTACCCTACCAAGG 540
DQ178602        CCACACCAGATAGCTCAGCTTGAGGCGACGACTTCCATTGCGCTGCGTACCCTACCAAGG 540
```

```
DQ267479        CCACACCAGATAGCTCAGCTTGAGGCGACGACTTCCATTGCGCTGCGTACCCTACCAAGG 540
JQ867073        CCACACCAGATAGCTCAGCTTGAGGCGACGACTTCCATTGCGCTGCGTACCCTACCAACG 540
X87232          CCACACCAGATAGCTCAGCTTGAGGCCACGAATTCCATTGCGCTGCGTACCCTACCAACG 540
JQ859916        CCACACCAGATAGCTCAGCTTGAGGCGACGACTTCCATTGCGCTGCGTACCCTACCAACG 540
                DQ148297_FW ──────►
DQ148297        CCACTCCAGCTAGCTCACCTTAAAGCGACGGCTTCCATTGCGCTGCGTACCCTACCAATG 537
X03103          CCACACCAGATAGCTCAGCTTGAGGCGACGACTTCCATTGCGCTGCGTACCCTACCAATG 540
JQ867086        CCACACCAAATAGCTCAACTTGAGGCGACAACTTCCATTGCGCTGCGTACCCTACCAACG 537
JQ867087        CCACACCAAATAGCTCAACTTGAGGCGACAACTTCCATTGCGCTGCGTACCCTACCAACG 537
JQ867089        CCACACCAAATAGCTCAACTTGAGGCGACAACTTCCATTGCGCTGCGTACCCTACCAACG 537
JQ867085        CCACACCAAATAGCTCAACTTGAGGCGACAACTTCCATTGCGCTGCGTACCCTACCAACG 537
GQ342970        CCACACCAAATAGCTCAACTTGAGGCGACAGCTTCCATTGCGCTGCGTACCCTACCAACG 537
GQ342971        CCACACCAAATAGCTCAACTTGAGGCGACAGCTTCCATTGCGCTGCGTACCCTACCAACG 537
DQ267478        CCACACCAAATAGCTCAACTTGAGGCGACAGCTTCCATTGCGCTGCGTACCCTACCAACG 537
GQ342972        CCACACCAAATAGCTCAACTTGAGGCGACAGTTTCCATTGCGCTGCGTACCCTACCAACG 537
GQ342976        CCACACCAAATAGCTCAACTTGAGGCGACAGCTTCCATTGCGCTGCGTACCCTACCAACG 537
                              ◄──── B3c_RV
GQ342973        CCACACCAGCTAGCTCAGCTTGAGGCGACGGCTTCCATTGCGCTGCGTACCCTACCAATG 537
GQ342975        CCACACCAGCTAGCTCAGCTTGAGGCGACGGCTTCCATTGCGCTGCGTACCCTACCAATG 537
                      ◄──── JQ867088_RV
JQ867088        CCACTCCAGCTAGCTCAGCTTGAAGCGACGGCTTCCATTGCGCTGCGTACCCTACCAATG 537
DQ826387        CCACACCAGATAGCTCAGTTTGAGGCGACGACTTCCATTGCGCTGCGTACCCTACCAACG 540
                **** ***  *******  ** * ** **   ************************** *

JQ859917        ATGTGCAATGTTAATGTGCCATTGTACGACATCATGCCACCCGACTTTTGGCACTAG--- 597
JQ867075        ATGTGCAATGTTAATGTGCCATTGTACGACATCATGCCACCCGACTTTTGGCACTAG--- 597
JQ867074        ATGTGCAATGTTAATGTGCCATTGTACGACATCATGCCACCCGACTTTTGGCACTAG--- 597
JQ859915        ATGTGCAATGTTAATGTGCCATTGTACGACATCATGCCGCCCGACTTTTGGCACTAG--- 597
X53690          ATGTGCAATGTTAATGTGCCATTGTACGACATCATGCCACCCGACTTTTGGCACTAG--- 597
JQ867081        ATGTGCAATGTTAATGTGCCATTGTACGACATCATGCCACCCGACTTTTGGCACTAG--- 597
JQ867083        ATGTGCAATGTTAATGTGCCATTGTACGACATCATGCCACCCGACTTTTGGCACTAG--- 597
JQ867082        ATGTGCAATGTTAATGTGCCATTGTACGACATCATGCCACCCGACTTTTGGCACTAG--- 597
JQ867084        ATGTGCAATGTTAATGTGCCATTGTACGACATCATGCCACCCGACTTTTGGCACTAG--- 597
DQ178602        ATGTGCAATGTTAATGTGCCATTGTACGACATCATGCCAGTCGACTTTTGGCACTAG--- 597
DQ267479        ATGTGCAATGTTAATGTGCCATTGTACGACATCATGCCAGTCGACTTTTGGCACTAG--- 597
JQ867073        ATGTGCAATGTTAATGTGCCATTGTATGACATCATGCCATTCGGCGTT-GGCACTAGAGT 599
X87232          ATGTGCAATGTTAATGTGCCATTGTATGACATCATGCCATTCGGCGTT-GGCACTAGAGT 599
JQ859916        ATGTGCAATGTTAATGTGCCATTGTATGACATCATGCCATTCGGCGTT-GGCACTAGAGT 599
                                                ◄──── DQ148297_RV
DQ148297        ATGTGTAGTGTTAATGTGCCGTTCTACCGCATACTTCCATTCGGCATT-GACACGAGAGT 596
X03103          ATGTGCAGTGTTAATGTGCCGTTGTACAGGATCCTGCGA---GGCGTT-GGCCCCAGTGT 596
JQ867086        ATGTGCAGTGTTAATGTGCCGTTGTACCGCATAGTGCCATTAGCCATT-GACACCAGAGT 596
JQ867087        ATGTGCAGTGTTAATGTGCCGTTGTACCGCATAGTGCCATTAGCCATT-GACACCAGAGT 596
JQ867089        ATGTGTAGTGTTAATGTGCCGTTGTACCGCATAGTGCCATTAGCCATT-GACACCAGAGT 596
JQ867085        ATGTGCAGTGTTAATGTGCCGTTGTACCGCATAGTGCCATTAGCCATT-GACACCAGAGT 596
GQ342970        ATGTGCAGTGTTAATGTGCCGTTGTACCGCATAGTGCCATTAGCCATT-GACACCAGAGT 596
GQ342971        ATGTGCAGTGTTAATGTGCCGTTGTACCGCATAGTGCCATTAGCCATT-GACACCAGAGT 596
DQ267478        ATGTGCAGTGTTAATGTGCCGTTGTACCGCATAGTGCCATTAGCCATT-GACACCAGAGT 596
GQ342972        ATGTGCAGTGTTAATGTGCCGTTGTACCGCATAGTGCCATTAGCCATT-GACACCAGAGT 596
GQ342976        ATGTGCAGTGTTAATGTGCCGTTGTACCGCATAGTGTCATTAGCCATT-GACACCAGAGT 596
GQ342973        ATGTGCAGTGTTAATCTGCCGTTGTACCGCATACTTCCATTCGGCATT-GACACGAGAGT 596
GQ342975        ATGTGCAGTGTTAATCTGCCGTTGTACCGCATACTTCCATTCGGCATT-GACACGAGAGT 596
JQ867088        ATGTGCAGTGTTAATGTGCCGTTCTACCGCATACTTCCATTCGGCATT-GACACGAGAGT 596
                       ◄──── DQ826387_RV
DQ826387        ATGTGCAGTGTTAATGTGCCGTCGTACAGGATCCTGCGA---GGCGTT-GGTCCCAGTGT 596
                ***** * ******* **** *  **     **  *        * * ** *    * **

JQ859917        ----------
JQ867075        ----------
JQ867074        ----------
JQ859915        ----------
X53690          ----------
JQ867081        ----------
JQ867083        ----------
JQ867082        ----------
JQ867084        ----------
DQ178602        ----------
DQ267479        ----------
JQ867073        TGATGTCTAA 609
X87232          TGGTGTCTAA 609
```

```
JQ859916        TGATGTCTAA 609
DQ148297        TGGTGTCTAA 606
X03103          TGGTGTCTAA 606
JQ867086        TGGTGTCTAA 606
JQ867087        TGGTGTCTAA 606
JQ867089        TGGTGTCTAA 606
JQ867085        TGGTGTCTAA 606
GQ342970        TGGTGTCTAA 606
GQ342971        TGGTGTCTAA 606
DQ267478        TGGTGTCTAA 606
GQ342972        TGGTGTCTAA 606
GQ342976        TGGTGTCTAA 606
GQ342973        TGGTGTCTAA 606
GQ342975        TGGTGTCTAA 606
JQ867088        TGGTGTCTAA 606
DQ826387        TGGTGTCTAA 606
```

C

```
X60037          ---------------ACCGCAAAAACCATTTCC-----AGTGC-AGCAACCGTTTCAC-- 37
JQ867090        CCTGCAACCCCAACCACCACAACAACCTTTTCCT---CAGTCCCAACAACCATTCAGCTG 57
S66938          ------------TCGGCCCCAACAACCATTCCCCTGGCAACCACAACAACCATTTCCC-- 46
                                ** *** **** ** **     *     * ***** **   *

X60037          ACA-CCCCAACAAT-ATTTCCCCTATCTACCAGAGGAATTGTTTCCCCAATATC---AAA 92
JQ867090        GCAACCACAACAACCATTTTCCCAGCC--CCAAC--AACCAGTTCCCCAACAACCACAAC 113
S66938          -CAGCCCCAACAACCAATT-CCCTACCAACCACAACAACCATTCAACCAGCAACCACAAC 104
                 ** ** ******  * ** ***   *  ***    **    *   ***  * *   **

X60037          TACCAACCCCCCTACAACCACAACAACCATTCCCCCAACAACCACAACAACCTCTTCCTC 152
JQ867090        AACCGTTCTCCCAGCAACCACAACAACCATTCCCCTGGCAACCACAACAACCATTTCCGC 173
S66938          AAATAATATCCCAGCAACCACAACAACCATTCCCCCAACAACCACAACAACCTTTTCCTC 164
                 *       ***  *********************   **************  **** *

X60037          GGCCCCAACAACCATTCCCCTGGCAACCACAACAACCATT-----TCCC----CAGCCCC 203
JQ867090        AGCCCCAACAACCAATTGCGCACCAACCACAACAACCATTCTCGTTCTCGCAGCAACCAC 233
                                                                      S66938_FW ---->
S66938          AGCCCCAACAACCATTCCCCTGGCAACCACAACAACCATT-----TCCC----CAGCCTC 215
                 ************* *  *    *****************     ** *    ** ** *

X60037          AAGAACCAATTCCCCAGCAACCACAACAACCATTCCCACAGCAACCACAACAACCATTCC 263
JQ867090        AACAACCATTCCCTCTGCAACCGCAACAATCATTCCCCCAACAACCACAACAACCATTTC 293
                                                         <---- S66938_RV
S66938          AACAACCATTTCCCCTGCAACCACAACAACCATTCCCCTGGCAACCACAACAACCATTTC 275
                ** ***** * ** * ****** ****** *******    ***************** *

X60037          CACAGCAACCACAACAAATAATTTTCCAGCAACCCCAACAATCATACCCTGT------GC 317
JQ867090        CCCAACAACCACAACAAATAATTTTCCAGCAACCCCAACAATCATACCCTGT------GC 347
S66938          C---GCAGCCCCAACAACCTATTGCGCACCAACCACAACAACCATTCTCGTTCTCGCAGC 332
                *    ** ** ******   ***   ** ***** ****** *** * *  *      **

                                           C1_FW --->
X60037          AACCTCAACAGCCATTTCCTC---AACCTCAAC---CAGTCCCCCAGCAGCGACCCCAAC 371
JQ867090        AACCTCAACAGCCATTTCCTC---AACCTCAAC---CAGTCCCCCAGCAGCGACCCCAAC 401
S66938          AACCACAACAACCATTCCCTCTGCAACCGCAACAACCATTCCCCCAACA---ACCACAAC 389
                **** ***** ***** ****   **** ****   ** ******* **   *** ****

                                   <---- C1_RV
X60037          AAGCATCCCCCCTACAACCGCAACCCCAACAAGCATCCCCCCTACAACCGCAACAACCAT 431
JQ867090        AAGCATCCCCCCTACAACCGCAAC------------------------------AACCAT 431
S66938          AACCATTTCCCCAACAACCACAAC------------------------------AAATAA 419
                ** ***  **** ****** ****                              **  *
```

```
X60037          TTCCCCAGGGATCAGAACAA--ATAATT-------CCCCAACAACCATTCCCTCTGCAAC 482
JQ867090        TTCCCCAGGGATCAGAACAA--ATAATT-------CCCCAACAACCATTCCCTCTGCAAC 482
S66938          TTTTCCAGCAACCCCAACAATCATACCCTGTGCAACCTCAACAGCCATTTCCTC---AAC 476
                **  ****  * *  *****  ***          ** ***** ***** ****   ***

X60037          CACAACCATTCCCCCAACAACCAC---AACAACCATTGCCCC---AGCCCCAACAACCAT 536
JQ867090        CACAACCATTCCCCCAACAACCAC---AACAACCATTGCCCC---AGCCCCAACAACCAT 536
S66938          CCCAACCAGTCCCCCAGCAACGACCCCAACAAGCATCCCCCCTACAACCGCAACAACCAT 536
                * ****** ******* **** **   ***** ***  ****   * ** **********

                                    Common C_FW——►
X60037          TCCGCCAACAAGCAGAACTAATAATTCCCCAGCAACCTCAACAACCATTGCCTCTGCAGC 596
JQ867090        TCCGCCAACAAGCAGAACTAATAATTCCCCAGCAACCTCAACAACCATTGCCTCTGCAGC 596
S66938          TTCCCCAGGGATCAGAACAAATAATTCCCCAGCAACCTCAACAACCATTCCCTCTGCAGC 596
                * * ***   * ****** ****************************** **********

                                ◄—— Common C_RV
X60037          CACACCAACCATATACACAACAAACCATCTGGAGTATGGTCTAG 640
JQ867090        CACACCAACCATATACACAACAAACCATCTGGAGTATGGTCTAG 640
S66938          CACACCAACCATATACACAACAAACCATCTGGAGTATGGTCTAG 640
                ********************************************
```

D

```
JQ867076        ------------------------------------------------------------
JQ867091        ------------------------------------------------------------
AY268139        ------------------------------------------------------------
JQ867077        ------------------------------------------------------------
D82941          AAGGGCAACAACCAGGACAAGGGCAACAAGGGTACCCAAGTGCAACTTTTCCACAACAGC 60

JQ867076        ------------------------------------------------------------
JQ867091        ------------------------------------------------------------
AY268139        ------------------------------------------------------------
JQ867077        CAGGACAATGGCAACAAGGGTCCTACCCAAGTACAACTTCTCCGCAGCAGTCAGGACAAG 60
D82941          CAGGACAATGGCAACAAGGGTCCTACCCAAGTACAACTTCTCCGCAGCAGTCAGGACAAG 120

JQ867076        GGCAACAAGGGTACAACCCAAGTGGAACTTCTACGCAGCAGCCGGGACAAGTGCAACAGT 60
JQ867091        GGCAACAAGGGTACAACCCAAGTGGAACTTCTACGCAGCAGCCGGGACAAGTGCAACAGT 60
AY268139        ------------------------------CTACGCAGCAGCCGGGACAAGTGCAACAGT 30
JQ867077        GGCAACAAGGGTACAACCCAAGTGGAACTTCTACGCAGCAGCCGGGACAAGTGCAACAGT 120
D82941          GGCAACAAGGGTACAACCCAAGTGGAACTTCTACGCAGCAGCCGGGACAAGTGCAACAGT 180
                                              ******************************

JQ867076        TGGGACAAGGGCAACAAGGGTACTACCCAATTGCAACTTCTCCGCAGCAGCCAGGACAAG 120
JQ867091        TGGGACAAGGGCAACAAGGGTACTACCCAATTGCAACTTCTCCGCAGCAGCCAGGACAAG 120
AY268139        TGGGACAAGGGCAACAAGGGTACTACCCAATTGCAACTTCTCCGCAGCAGCCAGGACAAG 90
JQ867077        TGGGACAAGGGCAACAAGGGTACTACCCAATTGCAACTTCTCCGCAGCAGCCAGGACAAG 180
D82941          TGGGACAAGGGCAACAAGGGTACTACCCAATTGCAACTTCTCCGCAGCAGCCAGGACAAG 240
                ************************************************************

JQ867076        GGCAACAGCTAGGACAAGGGCAACAACCAGGACATGGGCAACAGCTAGTGCAAGGGCAAC 180
JQ867091        GGCAACAGCTAGGACAAGGGCAACAACCAGGACATGGGCAACAGCTAGTGCAAGGGCAAC 180
AY268139        GGCAACAGCTAGGACAAGGGCAACAACCAGGACATGGGCAACAGCTAGTGCAAGGGCAAC 150
JQ867077        GGCAACAGCTAGGACAAGGGCAACAACCAGGACATGGGCAACAGCTAGTGCAAGGGCAAC 240
D82941          GGCAACAGCTAGGACAAGGGCAACAACCAGGACATGGGCAACAGCTAGTGCAAGGGCAAC 300
                ************************************************************

JQ867076        AACAAGGACAAGGGCAACAAGGACACTACCCAAGTATGACTTCTCCGCACCAAACAGGAC 240
JQ867091        AACAAGGACAAGGGCAACAAGGACACTACCCAAGTATGACTTCTCCGCACCAAACAGGAC 240
AY268139        AACAAGGACAAGGGCAACAAGGACACTACCCAAGTATGACTTCTCCGCACCAAACAGGAC 210
JQ867077        AACAAGGACAAGGGCAACAAGGACACTACCCAAGTATGACTTCTCCGCACCAAACAGGAC 300
D82941          AACAAGGACAAGGGCAACAAGGACACTACCCAAGTATGACTTCTCCGCACCAAACAGGAC 360
                ************************************************************

JQ867076        AAGGGCAAAAAGGATACTACCCAAGTGCAATTTCTCCGCAGCAGTCAGGACAAGGACAAC 300
JQ867091        AAGGGCAAAAAGGATACTACCCAAGTGCAATTTCTCCGCAGCAGTCAGGACAAGGACAAC 300
AY268139        AAGGGCAAAAAGGATACTACCCAAGTGCAATTTCTCCGCAGCAGTCAGGACAAGGACAAC 270
JQ867077        AAGGGCAAAAAGGATACTACCCAAGTGCAATTTCTCCGCAGCAGTCAGGACAAGGACAAC 360
```

```
D82941          AAGGGCAAAAAGGATACTACCCAAGTGCAATTTCTCCGCAGCAGTCAGGACAAGGACAAC 420
                ************************************************************

JQ867076        AAGGATACCAGCCTAGTGGAGCTTCTTCACAGGGGTCGGTGCAAGGGGCGTGCCAGCACA 360
JQ867091        AAGGATACCAGCCTAGTGGAGCTTCTTCACAGGGGTCGGTGCAAGGGGCGTGCCAGCACA 360
AY268139        AAGGATACCAGCCTAGTGGAGCTTCTTCACAGGGGTCGGTGCAAGGGGCGTGCCAGCACA 330
JQ867077        AAGGATACCAGCCTAGTGGAGCTTCTTCACAGGGGTCGGTGCAAGGGGCGTGCCAGCACA 420
D82941          AAGGATACCAGCCTAGTGGAGCTTCTTCACAGGGGTCGGTGCAAGGGGCGTGCCAGCACA 480
                ************************************************************

JQ867076        GCACATCTTCTCCGCAGCAGCAAGCACAAGGGTGCCAAGCTTCTTCACCAAAGCAAGGGC 420
JQ867091        GCACATCTTCTCCGCAGCAGCAAGCACAAGGGTGCCAAGCTTCTTCACCAAAGCAAGGGC 420
AY268139        GCACATCTTCTCCGCAGCAGCAAGCACAAGGGTGCCAAGCTTCTTCACCAAAGCAAGGGC 390
JQ867077        GCACATCTTCTCCGCAGCAGCAAGCACAAGGGTGCCAAGCTTCTTCACCAAAGCAAGGGC 480
D82941          GCACATCTTCTCCGCAGCAGCAAGCACAAGGGTGCCAAGCTTCTTCACCAAAGCAAGGGC 540
                ************************************************************

JQ867076        TAGGGTCGTTGTACTACCCGAGTGGAGCTTATACACAACAGAAACCAGGGCAAGGGTACA 480
JQ867091        TAGGGTCGTTGTACTACCCGAGTGGAGCTTATACACAACAGAAACCAGGGCAAGGGTACA 480
AY268139        TAGGGTCGTTGTACTACCCGAGTGGAGCTTATACACAACAGAAACCAGGGCAAGGGTACA 450
JQ867077        TAGGGTCGTTGTACTACCCGAGTGGAGCTTATACACAACAGAAACCAGGGCAAGGGTACA 540
D82941          TAGGGTCGTTGTACTACCCGAGTGGAGCTTATACACAACAGAAACCAGGGCAAGGGTACA 600
                ************************************************************

JQ867076        ACCCAGGTGGAACTTCTCCGCTGCACCAGCAAGGGGGAGGGTTCGGCGGCGGGTTAACGA 540
JQ867091        ACCCAGGTGGAACTTCTCCGCTGCACCAGCAAGGGGGAGGGTTCGGCGGCGGGTTAACGA 540
AY268139        ACCCAGGTGGAACTTCTCCGCTGCACCAGCAAGGGGGAGGGTTCGGCGGCGGGTTAACGA 510
JQ867077        ACCCAGGTGGAACTTCTCCGCTGCACCAGCAAGGGGGAGGGTTCGGCGGCGGGTTAACGA 600
D82941          ACCCAGGTGGAACTTCTCCGCTGCACCAGCAAGGGGGAGGGTTCGGCGGCGGGTTAACGA 660
                ************************************************************

JQ867076        CGGAGCAACCGCAGGGAGGAAAGCAGCCATTCCATTGCCAGCAAACCACTGTCTCCCCTC 600
JQ867091        CGGAGCAACCGCAGGGAGGAAAGCAGCCATTCCATTGCCAGCAAACCACTGTCTCCCCTC 600
AY268139        CGGAGCAACCGCAGGGAGGAAAGCAGCCATTCCATTGCCAGCAAACCACTGTCTCCCCTC 570
JQ867077        CGGAGCAACCGCAGGGAGGAAAGCAGCCATTCCATTGCCAGCAAACCACTGTCTCCCCTC 660
D82941          CGGAGCAACCGCAGGGAGGAAAGCAGCCATTCCATTGCCAGCAAACCACTGTCTCCCCTC 720
                ************************************************************

JQ867076        ACCAGGGTCAGCAAACCACTGTTTCCCCTCATCAGGGTCAGCAAACCACTGTCTCCCCTC 660
JQ867091        ACCGGGGTCAGCAAACCACTGTTTCCCCTCATCAGGGTCAGCAAACCACTGTCTCCCCTC 660
AY268139        ACCAGGGTCAGCAAACCACTGTCTCCCCTCATCAGGGTCAGCAAACCACTGTCTCCCCTC 630
JQ867077        ACCAGGGTCAGCAAACCACTGTTTCCCCTCATCAGGGTCAGCAAACCACTGTCTCCCCTC 720
D82941          ACCAGGGTCAGCAAACCACTGTTTCCCCTCATCAGGGTCAGCAAACCACTGTCTCCCCTC 780
                *** ****************** *************************************

JQ867076        ATCAGGGTCAGCAAACCACTGTCTCCCCTCACCAGGGTCAGCAAACCACCGTCTCCCCTC 720
JQ867091        ATCAGGGTCAGCAAACCACTGTCTCCCCTCACCAGGGTCAGCAAACCACCGTCTCCCCTC 720
AY268139        ATCAGGGTCAGCAAACCACGGTCTCCCCTCACCAGGGTCAGCAAACCACCGTCTCCCCTC 690
JQ867077        ATCAGGGTCAGCAAACCAC----------------------------------------- 739
D82941          ATCAGGGTCAGCAAACCAC----------------------------------------- 799
                *******************

JQ867076        ACCAGGGTCAGCAAACCACCGTCTCCCCTCATCAGGGTCAGCAAACCACTGTCTCCCCTC 780
JQ867091        ACCAGGGTCAGCAAACCACCGTCTCCCCTCATCAGGGTCAGCAAACCACTGTCTCCCCTC 780
AY268139        ACCAGGGTCAGCAAACCACCGTCTCCCCTCATCAGGGTCAGCAAACCACTGTCTCCCCTC 750
JQ867077        -------------------CGTCTCCCCTCATCAGGGTCAGCAAACCACTGTCTCCCCTC 780
D82941          -------------------TGTCTCCCCTCACCAGGGTCAGCAAACCACCGTCTCCCCTC 840
                                    *********** ***************** **********

JQ867076        ATCCGGGTCAGCAAACCACTGTCTCCCCTCATCAGGGTCAGCAAACCACTGTCTCCCCTC 840
JQ867091        ATCCGGGTCAGCAAACCACTGTCTCCCCTCATCAGGGTCAGCAAACCACTGTCTCCCCTC 840
AY268139        ATCCGGGTCAGCAAACCACTGTCTCCCCTCATCAGGGTCAGCAAACCACTGTCTCCCCTC 810
JQ867077        ATCCGGGTCAGCAAACCACTGTCTCCCCTCATCAGGGTCAGCAAACCACTGTCTCCCCTC 840
D82941          ACCAGGGTCAGCAAACCACCGTCTCCCCTCATCAGGGTCAGCAAACCACTGTCTCCCCTC 900
                * * *************** ****************************************

JQ867076        ATCCGGGTCAGCAAACCACTGTCTCCCCTCATCAGGGTCAGCAAACCACTGTCTCCCCTC 900
JQ867091        ATCCGGGTCAGCAAACCACTGTCTCCCCTCATCAGGGTCAGCAAACCACTGTCTCCCCTC 900
AY268139        ATCCGGGTCAGCAAACCACTGTCTCCCCTCATCAGGGTCAGCAAACCACTGTCTCCCCTC 870
JQ867077        ATCCGGGTCAGCAAACCACTGTCTCCCCTCATCAGGGTCAGCAAACCACTGTCTCCCCTC 900
D82941          ATCCGGGTCAGCAAACCAC----------------------------------------- 919
```

```
                *******************

JQ867076        ATCAGGGTCAGCAAACCAC------------------------------CGTCTCCCCTC 930
JQ867091        ATCAGGGTCAGCAAACCAC------------------------------CGTCTCCCCTC 930
AY268139        ATCAGGGTCAGCAAACCACTGTCTCCCCTCATCAGGGTCAGCAAACCACCGTCTCCCCTC 930
JQ867077        ATCAGGGTCAGCAAACCAC------------------------------CGTCTCCCCTC 930
D82941          -------------------------------------------------CGTCTCCCCTC 930
                                                                 ***********

JQ867076        ATCAGGGTCAGCAAACCACCGTCTCCCCTCATCAGGGTCAGCAAACCACCGTCTCCCCTC 990
JQ867091        ATCAGGGTCAGCAAACCACCGTCTCCCCTCATCAGGGTCAGCAAACCACCGTCTCCCCTC 990
AY268139        ATCAGGGTCAGCAAACCACCGTCTCCCCTCATCAGGGTCAGCAAACCACCGTCTCCCCTC 990
JQ867077        ATCAGGGTCAGCAAACCACCGTCTCCCCTCATCAGGGTCAGCAAACCACCGTCTCCCCTC 990
D82941          ATCAGGGTCAGCAAACCACCGTCTCCCCTCATCAGGGTCAGCAAACCACCGTCTCCCCTC 990
                ************************************************************
                                                                    Common D_FW →
JQ867076        ATCAGGGTCAGCAGCCCGGCGAGCAGCCTTGCGGTTTCCCTGGCCAGCAAACCACCGTGT 1050
JQ867091        ATCAGGGTCAGCAGCCCGGCGAGCAGCCTTGCGGTTTCCCTGGCCAGCAAACCACCGTGT 1050
AY268139        ATCAGGGTCAGCAGCCCGGCGAGCAGCCTTGCGGTTTCCCTGGCCAGCAAACCACCGTGT 1050
JQ867077        ATCAGGGTCAGCAGCCCGGCGAGCAGCCTTGCGGTTTCCCTGGCCAGCAAACCACCGTGT 1050
D82941          ATCAGGGTCAGCAGCCCGGCGAGCAGCCTTGCGGTTTCCCTGGCCAGCAAACCACCGTGT 1050
                ************************************************************
                                          ← Common D_RV
JQ867076        CTCTGCACCATGGTCAGCAGTCCAACGAGTTGTACTACGGCAGCCCATACCATGTTAGCG 1110
JQ867091        CTCTGCACCATGGTCAGCAGTCCAACGAGTTGTACTACGGCAGCCCATACCATGTTAGCG 1110
AY268139        CTCTGCACCATGGTCAGCAGTCCAACGAGTTGTACTACGGCAGCCCATACCATGTTAGCG 1110
JQ867077        CTCTGCACCATGGTCAGCAGTCCAACGAGTTGTACTACGGCAGCCCATACCATGTTAGCG 1110
D82941          CTCTGCACCATGGTCAGCAGTCCAACGAGTTGTACTACGGCAGCCCATACCATGTTAGCG 1110
                ************************************************************

JQ867076        TGGAGCAGCCGTCGGCCAGCCTAAAGGTAGCAAAGGCGCAGCAGCTCGCGGCGCAGCTGC 1170
JQ867091        TGGAGCAGCCGTCGGCCAGCCTAAAGGTAGCAAAGGCGCAGCAGCTCGCGGCGCAGCTGC 1170
AY268139        TGGAGCAGCCGTCGGCCAGCCTAAAGGTAGCAAAGGCGCAGCAGCTCGCGGCGCAGCTGC 1170
JQ867077        TGGAGCAGCCGTCGGCCAGCCTAAAGGTAGCAAAGGCGCAGCAGCTCGCGGCGCAGCTGC 1170
D82941          TGGAGCAGCCGTCGGCCAGCCTAAAGGTAGCAAAGGCGCAGCAGCTCGCGGCGCAGCTGC 1170
                ************************************************************

JQ867076        CGGCAATGTGTCGGCTGGAGGGCGGCGGCGGCCTGTTGGCCAGCCAGTAG 1220
JQ867091        CGGCAATGTGCCGGCTGGAGGGCGGCGGCGGCCTGTTGGCCAGCCAGTAG 1220
AY268139        CGGCAATGTGTCGGCTGGAGGGCGGCGGCGGCCTGTTGGCCAGCCAGTAG 1220
JQ867077        CGGCAATGTGTCGGCTGGAGGGCGGCGGCGGCCTGTTGGCCAGCCAGTAG 1220
D82941          CGGCAATGTGTCGGCTGGAGGGCGGCGGCGGCCTGTTGGCCAGCCAGTAG 1220
                ********** ***************************************
```

γ

```
JQ867078        CCAGCGGCCACTAC---TACCATTTACTCACCCGTTCCTAACATTCCCCGACCAACTACT 57
X13508          CCAACGGCCACTAC---TACCATTTACTCACCCGTTCCTAACATTCCCCGACCAACTACT 57
AJ580585        CCAACGGCCACTAC---TACCATTTACTCACCCGTTCCTAACATTCCCCGACCAACTACT 57
JQ867079        CCAACGGCCACTAC---TACCGTTTACCCACCCGTTCCTACCATTCCCCGACCAACTACT 57
X72628          ACAATTTCCGCAGCAGATGCCACT---CCAGCCGCAACAACAATTTCCGCAGCAGATGCC 57
JQ867080        TCAATTTCCACAGCAGCTGCCAC------------AACAACAATTTCCGCAGCAGATGCC 48
                 **    ** *  *   * **                * *  *** **  * **  * *

JQ867078        CCCTCAGCCACCACACCAATCATTCCCCCAGCCCCCGCAATCATACCC--TCAGCCACCG 115
X13508          CCCTCAGCCACCACACCAATCATTCCCCCAGCCCCCACAATCATACCC--TCAGCCACCG 115
AJ580585        CCCTCAGCCACCACACCAATCATTCCCCCAGCCCCCGCAATCATACCC--TCAGCCACCG 115
JQ867079        CCCTCAGCCACCACACCAATCATTCCCCCAGCCCCCACAATCATACCC--TCAGCCACCG 115
X72628          ACTCCAGCCGCAACAACAACCA------CAATTCCCGCAACAAAAACCATTTGGCCAATA 111
JQ867080        ACTCCAGCCGCAACAACAACCA------CAATTCCCGCAACAAAAACCATTTGGCCAATA 102
                 *  ***** * *** *** **      **   *** ***  * * **  *  ****

JQ867078        TTA-CAACCTTTTCCTCAGCCCCCGCAACAAAAATATCCCGAGCAACCACAACAACCATT 174
X13508          TTA-CAACCTTTTCCTCAGCCCCCGCAACAAAAATATCCCGAGCAACCACAACAACCATT 174
AJ580585        TTA-CAACCTTTTCCTCAGCCCCCGCAACAAAAATATCCCGAGCAACCACAACAACCATT 174
JQ867079        TTA-CAACCTTTTCCTCAGCCCCCGCAACAAAAATATCCCGAGCAACCACAACAACCATT 174
X72628          TCAACAACCATT-----AA----CACAACAACCATATCC---GCA---ACAGCAACCATT 156
JQ867080        TCAACAACCATT-----AA----CACAACAACCATATCC---GCA---ACAGCAACCATT 147
```

```
                * * ***** **      *      * ******  ******    ***    *** ********

JQ867078        TCCTTGGCAACAACCAACTATTCAGCTATATCTCCAACAACAACTGAATCCGTGCAAGGA 234
X13508          TCCTTGGCAACAACCAACTATTCAGCTATATCTCCAACAACAACTGAATCCGTGCAAGGA 234
                                                 AJ580585_FW——>
AJ580585        TCCTTGGCAACAACCAACTATTCAGCTATATCTCCAACAACAACTGAATCCGTACAAGGA 234
JQ867079        TCCTTGGCAACAACCAACTATTCAGCTATATCTCCAACAACAACTGAATTCGTGCAAGGA 234
X72628          GGCACAACAACAACCTTCTATAGAGGAA------CAACATCAATTGAACTTGTGCAAGGA 210
JQ867080        GGCACAACAACAACCTTCTATAGAGGAA------CAACATCAATTGAACTTGTGCAAGGA 201
                  *     ********  ****   **   *       ***** *** ****    ** ******

JQ867078        GTTTCTCTTGCAACAGTGCA-----GGC---------CTGT-------GTCATTGTTATC 273
                                          X13508_FW——>
X13508          GTTTCTCTTGCAACAGTGCA-----GGC---------CTGT-------GTCATTGTTATC 273
AJ580585        GTTTCTCTTGCAACAGTGCA-----GGC---------CTGT-------GTCATTGTTATC 273
JQ867079        GTTTCTCTTGCAACAGTGCA-----GGC---------CTGT-------GTCATTGTTATC 273
X72628          GTTCCTCCTACAGCAGTGCACCCTGGACGAGAAGGTACCATTACTCCAGTCAGTGATCTC 270
JQ867080        GTTCCTCCTACAACAGTGCACCCTGGACGAGAAGGTACCATTACTCCAGTCAGTGATCTC 261
                *** *** * ** *******     * *           *  *        **** ** * **

JQ867078        GTACCTCTGGTCAAAGATCGTGCAACAGAGCAGTTGTCGGGTGATGCAGCAACAATGTTG 333
                                        <——X13508_RV
X13508          GTACATCTGGTCAAAGATCGTGCAACAGAGCAGTTGTCGGGTGATGCAGCAACAATGTTG 333
AJ580585        GTACCTCTGGTCAAAGATCGTGCAACAGAGCAGTTGTCGGGTGATGCTGCAACAATGTTG 333
JQ867079        GTACCTCTGGTCAAAGATCTTGCAACAGAGCAGTTGTCGGGTGATGCAGCAACAGTGTTG 333
X72628          GTTCCTCCGACCACATATCTCGCAACAAAACAGTTGCCAATTGAAGCGACAACAATGTTG 330
JQ867080        GTTCCTCCGACCACATATCTCGCAACAAAACAGTTGCCAATTGAAGCGACAACAATGTTG 321
                ** * ** *  ** * ***  ****** * ****** *    *** **   ***** *****

JQ867078        CCTGCAATTGGCACAGATCCCTGAACAATACAAATGCACAGCAATCGATAGCATTGTGCA 393
X13508          CCTGCAATTGGCACAGATCCCTGAACAATACAAATGCACAGCAATCGATAGCATTGTGCA 393
        <——AJ580585_RV
AJ580585        CCTGCAATTGGCACAGATCCCTGAACAATACAAATGCACAGCAATCGATAGCATTGTGCA 393
JQ867079        CCTGCAATTGGCACAGATCCCTGAACAATACAAATGCACAGCAATTGATAGCATTGTGCA 393
                          X72628_FW——>
X72628          TCAACAACTAGCAAATATCAATGAGCAGTCCCGATGCCCAGCCATCCAAACCATTGTGCA 390
                          JQ867080_FW——>
JQ867080        TCAACAACTAGCAAATATCAATGAGCAATCCCGATGCCCAGCCATCCAAACCATTGTGCA 381
                 *  *** * *** * ***  *** ** * *  **** **** **   * * *********

JQ867078        TGCCATCTTCATGCAGCAAGGCCAA------AGACAA-GGCGTGCAGATCGTGCAACAA- 445
X13508          TGCCATCTTCATGCAGCAAGGCCAA------AGACAA-GGCGTGCAGATCGTGCAACAA- 445
AJ580585        TGCCATCTTCATGCAGCAAGGCCAA------AGACAA-GGCGTGCAGATCGTGCAACAA- 445
JQ867079        TGCCATCTTCATGCAGCAAGGCCAA------AGACAA-GGCGTGCAGATCGTGCAACAA- 445
X72628          TGCCATCGTCATGCAACAACAA---------GTACAACAACAGGTGGGTCATGGTTTTGT 441
JQ867080        TGCCATCGTCATGCAACAACAACAACAACAAGTACAACAACAGGTGGATCATGGTTTTGT 441
                ******* ******* ***                ****    *  *   * ** **

JQ867078        -CAACCTCAGCCGCAACAGGTGGGTCAATGTGTGCTCGTCCAG---------------GG 489
X13508          -CAACCTCAGCCGCAACAGGTGGGTCAATGTGTGCTCGTCCAG---------------GG 489
AJ580585        -CAACCTCAGCCGCAACAGGTGGGTCAATGTGTGCTCGTCCAG---------------GG 489
JQ867079        -CAACCTCAGCCGCAACAGGTGGGTCAATGTGTACTCGTCCAG---------------GG 489
       <——X72628_RV
X72628          CCAATCTCAACTGCAACAGTTGGGCCAGGGAATGCCCATCCAGCTTCAACAACAGCCGGG 501
     <——JQ867080_RV
JQ867080        CCAATCTCAACTGCAACAGTTGGGCCAGGGAATGCCCATCCAGCTTCAACAACAGCCGGG 501
                 *** **** * ******* **** **   *   * * * *****                **

                              Common γ1_FW——>
JQ867078        TCAGGGCGTCGTCCAACCGCAACAACTAGCTCAAATGGAAGCAATCAGAACGTTGGTGTT 549
X13508          TCAGGGCGTCGTCCAACCGCAACAACTAGCTCAAATGGAAGCAATCAGAACGTTGGTGTT 549
AJ580585        TCAGGGCGTCGTCCAACCGCAACAACTAGCTCAAATGGAAGCAATCAGAACGTTGGTGTT 549
JQ867079        TCAGGGCGTCGCCCAACCGCAACAACTAGCTCAAATGGAAGCAATCAGAACGTTGGTGTT 549
                                                          Common γ3_FW——>
X72628          CCAGGCCTTTGTCCTACCTCAACAACAAGCCCAGTTCAAGGTGGTTGGGTCATTGGTGAT 561
JQ867080        CCAGGCCTTTGTCCTACCTCAACAACAAGCCCAGTTCAAGGTGGTTGGGTCATTGGTGAT 561
                 **** * * * ** *** ******* *** **   *  * *    *  *  * ****** *

JQ867078        GCAGTCCGTTCCATCCATGTGCAACTTTAATGTCCCACCTAACTGCTCCACCATCAAAGC 609
```

```
X13508          GCAGTCCGTTCCATCCATGTGCAACTTTAATGTCCCACCTAACTGCTCCACCATCAAAGC 609
AJ580585        GCAGTCCGTTCCATCCATGTGCAACTTTAATGTCCCACCTAACTGCTCCACCATCAAAGC 609
JQ867079        GCAGTCCGTTCCATCCATGTGCAACTTTAATGTCCCACCTAACTGCTCCACCATCAAAGC 609
                                              ←—— Common γ3_RV
X72628          TCAGACACTTCCTATGTTATGCAATGTGCATGTCCCACCTTATTGCT------------C 609
JQ867080        TCAGACACTTCCTATGTTATGCAATGTGCATGTCCCACCTTATTGCT------------C 609
                 *** *  ****     * *****  *  *********** * ****            *

            ←—— Common γ1_RV
JQ867078        ACCATTTGTTGGTGTAGTCACCGGTGTTGGAGGACAATGA 649
X13508          ACCATTTGTTGGTGTAGTCACCGGTGTTGGAGGACAATGA 649
AJ580585        ACCATTTGTTGGTGTAGTCACCGGTGTTGGAGGACAATAA 649
JQ867079        ACCATTTGTTGGTGTAGTCACCGGTGTTGGAGGACAATGA 649
X72628          ACCATTTGGTAGCATGGCTACCGGCAGTGGTGGCCAATGA 649
JQ867080        ACCATTTGGTAGCATGGCTACCGGCAGTGGTGGCCAATGA 649
                ******** * *  * *  *****   *** ** **** *
```
