# Supplementary material for: Quantitative RT-PCR based platform for rapid quantification of the transcripts of highly homologous multigene families and their members during grain development
Source: BMC Plant Biol. 2012 Oct 9;12:184. doi: 10.1186/1471-2229-12-184 (PMC3492166; doi:10.1186/1471-2229-12-184)
Supplement: Additional file 2 — Abundance of hordein groups during grain development using actin for normalisation. [file 1471-2229-12-184-S2.pdf]

**Primer**	**Normalised amount of hordein group (amol of hordein/amol of actin)**			
	**10 DAP**	**15 DAP**	**18 DAP**	**25 DAP**
**Common B**	341.55	2239.68	1773.93	2695.45
B1	144.35	915.85	638.80	530.90
B1a	ND	ND	ND	ND
X87232	50.67	319.79	221.24	249.83
JQ867084	ND	ND	ND	ND
DQ148297	ND	ND	ND	ND
B2	1.64	10.38	8.84	6.24
B3	144.82	616.98	624.04	1131.05
B3a	0.83	3.69	3.72	3.10
B3b	85.56	399.93	409.81	930.36
B3c	ND	ND	ND	ND
DQ826387	0.78	8.03	10.24	33.10
GQ342976	0.20	0.40	0.35	0.81
JQ867088	ND	ND	ND	ND
**Common C**	23.62	135.18	144.92	292.33
C1	0.21	2.07	2.49	6.16
S66938	15.87	119.96	111.61	191.89
**Common D**	6.37	67.42	54.44	63.46
**Common γ1**	16.58	48.68	64.78	52.50
AJ580585	ND	ND	ND	ND
X13508	ND	ND	ND	ND
**Common γ3**	24.07	130.53	160.44	247.57
X72628	ND	ND	ND	ND
JQ867080	ND	ND	ND	ND

ND- not detected
